# Efficacy of novel bacterial consortia in degrading fipronil and thiobencarb in paddy soil: a survey for community structure and metabolic pathways

**DOI:** 10.3389/fmicb.2024.1366951

**Published:** 2024-05-15

**Authors:** Nastaran Faridy, Ehssan Torabi, Ahmad Ali Pourbabaee, Ebrahim Osdaghi, Khalil Talebi

**Affiliations:** ^1^Department of Plant Protection, Faculty of Agriculture, College of Agriculture and Natural Resources, University of Tehran, Karaj, Iran; ^2^Department of Soil Science, Faculty of Agriculture, College of Agriculture and Natural Resources, University of Tehran, Karaj, Iran

**Keywords:** pesticide, bioremediation, response surface methodology, soil bioaugmentation, transformation products

## Abstract

**Introduction:**

Fipronil (FIP) and thiobencarb (THIO) represent widely utilized pesticides in paddy fields, presenting environmental challenges that necessitate effective remediation approaches. Despite the recognized need, exploring bacterial consortia efficiently degrading FIP and THIO remains limited.

**Methods:**

This study isolated three unique bacterial consortia—FD, TD, and MD—demonstrating the capability to degrade FIP, THIO, and an FIP + THIO mixture within a 10-day timeframe. Furthermore, the bioaugmentation abilities of the selected consortia were evaluated in paddy soils under various conditions.

**Results:**

Sequencing results shed light on the consortia’s composition, revealing a diverse bacterial population prominently featuring *Azospirillum*, *Ochrobactrum*, *Sphingobium*, and *Sphingomonas* genera. All consortia efficiently degraded pesticides at 800 µg/mL concentrations, primarily through oxidative and hydrolytic processes. This metabolic activity yields more hydrophilic metabolites, including 4-(Trifluoromethyl)-phenol and 1,4-Benzenediol, 2-methyl-, for FIP, and carbamothioic acid, diethyl-, S-ethyl ester, and Benzenecarbothioic acid, S-methyl ester for THIO. Soil bioaugmentation tests highlight the consortia’s effectiveness, showcasing accelerated degradation of FIP and THIO—individually or in a mixture—by 1.3 to 13-fold. These assessments encompass diverse soil moisture levels (20 and 100% *v/v*), pesticide concentrations (15 and 150 µg/g), and sterile conditions (sterile and non-sterile soils).

**Discussion:**

This study offers an understanding of bacterial communities adept at degrading FIP and THIO, introducing FD, TD, and MD consortia as promising contenders for bioremediation endeavors.

## 1 Introduction

In contemporary agriculture, the predominant strategy for pest management in agroecosystems involves the widespread use of pesticides (Topping et al., 2020). While effective in controlling pests, the extensive application of pesticides raises concerns about environmental pollution, affecting soil, sediments, and ground and surface waters. This issue is surged by the potential bioaccumulation of pesticides in the food chain, posing risks to human health (Lin et al., 2020; Pang et al., 2020).

Fipronil (FIP), an insecticide from the phenylpyrazole chemical family, is commonly employed in rice paddies to combat the rice stem borer, *Chilo suppressalis* (Lepidoptera: Crambidae). FIP tends to persist in soil, with a soil sorption coefficient (K_oc_) ranging from 825 to 6,863 (Ying and Kookana, 2006; Lin et al., 2009), and it shows propensity to accumulate in non-target organisms such as fish and earthworms, as indicated by its bioconcentration factor (BCF) (Jackson et al., 2009; Qu et al., 2014). Classified as environmentally harmful, with an environmental impact quotient (EIQ) of 103.5 (Moinoddini et al., 2014), FIP also poses significant risks to honeybees and other beneficial insects (Zaluski et al., 2015; Kasai et al., 2016). Similarly, Thiobencarb (THIO), a systemic thiocarbamate herbicide used in paddies, exhibits moderate to high toxicity to aquatic organisms and has the potential to cause soil and groundwater pollution due to its moderate mobility in soil (Saka, 2010; Wang et al., 2019).

Considering the environmental repercussions of pesticide use, bioremediation utilizing soil microorganisms emerges as an effective approach to mitigate agrochemical pollution (Torabi et al., 2017; Pourbabaee A. et al., 2018; Pourbabaee A. A. et al., 2018; Pourbabaei et al., 2020; Li J. et al., 2022; Pang et al., 2023). Several indigenous microbial strains have been reported to mineralize FIP, including *Aspergillus* sp., *Bacillus thuringiensis*, *Klebsiella pneumoniae*, *Paracoccus* sp., *Staphylococcus arlettae*, *Stenotrophomonas* sp., and *Streptomyces* sp. (Kumar et al., 2012; Uniyal et al., 2016; Gajendiran and Abraham, 2017; Abraham and Gajendiran, 2019; Bhatt et al., 2021a, 2021b). Similarly, aerobic and anaerobic microbial strains capable of degrading THIO have been recorded in the literature, i.e., *Acidovorax* sp., *Aspergillus niger*, *Azoarcus* sp., *Corynebacterium* sp., *Cupriavidus oxalaticus*, *Dechloromonas* sp., *Pseudomonas* sp., and *Thauera* sp. (Miwa et al., 1988; Chu et al., 2017; Duc, 2023; Duc et al., 2023). However, the limitations of single-strain approaches, which may result in incomplete pesticide degradation and the formation of more toxic metabolites, necessitate a shift toward microbial community-level interventions (Bai et al., 2020; Li et al., 2020; Qin et al., 2020). Recent studies emphasize the functional roles of microbial consortia and unculturable microorganisms in environmental pollutant biodegradation, highlighting the superior degradation potential of mixed microorganisms through synergistic metabolism (Bai et al., 2020; Li et al., 2020; Qin et al., 2020; Zhang et al., 2020; Lin et al., 2022; Pang et al., 2023).

So far, there has been no report on the isolation of microbial consortia for the degradation of FIP and THIO. To address the existing knowledge gap, this research aims to (1) isolate and identify microbial consortia proficient in degrading FIP and THIO individually or in combination, (2) assess optimal degradation conditions for FIP and THIO by these consortia, (3) elucidate the metabolic pathways employed by the consortia in FIP and THIO degradation, and (4) evaluate the consortia’s efficiency in degrading FIP and THIO under various soil conditions. Through these objectives, we seek to contribute valuable insights into the potential application of microbial consortia for the bioremediation of FIP and THIO-contaminated environments.

## 2 Materials and methods

### 2.1 Chemicals and culture medium

Analytical grade FIP (≥ 95%) and THIO (≥ 98%) were procured from Sigma–Aldrich (Supplementary Table S1). All solvents used in this research, such as methanol (MeOH), acetone (Ace), and acetonitrile (MeCN), were of analytical grade quality (99.9%) and obtained from Merck, Germany. Anhydrous magnesium sulfate (MgSO_4_) and sodium chloride (NaCl) of reagent grade purity at 99% were supplied by BioShop, Burlington, Canada. Primary–secondary amine (PSA) was supplied by Agilent Technologies, United States. Stock solutions of FIP and THIO (1 g/L) were prepared in Ace and MeOH for soil/culture media spikings and chromatographic analyses, respectively. These solutions were stored in amber glass vials at −20°C to ensure stability.

A mineral salt medium (MSM) (Cycoń et al., 2009) composed of 2 g (NH_4_)_2_SO_4_, 0.2 g MgSO_4_.7H_2_O, 0.01 g CaCl_2_.2H_2_O, 0.001 g FeSO_4_.7H_2_O, 1.5 g Na_2_HPO_4_.12H_2_O, 1.5 g KH_2_PO_4_, and 0.5 g K_2_HPO_4_ per liter of deionized water was employed. The pH of the medium was adjusted to 7.2. Before usage, the medium was sterilized at 121°C for 20 min.

### 2.2 Soil sample collection and enrichment of FIP and THIO degrading consortia

Soil samples were collected from a paddy field in Amol County, Mazandaran Province, Northern Iran. The sampling location has long been known for its extensive use of FIP and THIO pesticides (Supplementary Table S2).

The enrichment process was carried out in 250 mL Erlenmeyer flasks, each containing 100 mL of the MSM medium. The medium was supplemented with FIP, THIO, or a combination of both pesticides (the mixture of FIP and THIO) at a concentration of 25 μg/mL, serving as the exclusive carbon source. Pesticide stock solutions in Ace were gently mixed into the flasks, followed by a 24-h incubation period to allow for Ace evaporation under sterile conditions. Subsequently, 5 g of air-dried soil was introduced to the flasks, and they were incubated in darkness at 30°C while being shaken at 120 rpm for 10 days. Following this incubation period, 5 mL of the content from each flask was transferred to fresh 100 mL MSM medium amended with pesticides at a concentration of 50 μg/mL. The same incubation conditions as mentioned above were maintained for an additional 10 days.

Throughout the incubation period, samples were periodically taken from each flask for FIP and THIO degradation and microbial growth measurements. High-performance liquid chromatography (HPLC) was employed to confirm pesticide degradation. At the same time, microbial growth was assessed by monitoring changes in the medium at an optical density of 600 nm (OD_600_) using a spectrophotometer. After several successive enrichment cycles at a concentration of 50 μg/mL, three consortia displaying the most satisfactory growth and efficiency in degrading FIP, THIO, and the FIP + THIO mixture within 7 days were selected and designated as FD, TD, and MD, respectively. These consortia were harvested by centrifugation at 2,124 × g and 4°C, followed by two rounds of washing with sterile phosphate-buffered saline (PBS) at pH 7.0. Subsequently, the consortia were resuspended in sterilized MSM, supplemented with 40% glycerin, resulting in a final cell concentration of approximately 10^8^ cells/ml. The inocula were then cryopreserved at −80°C for subsequent experiments and long-term usage (Xu et al., 2020; Pang et al., 2023).

### 2.3 Evaluation of consortia growth and degradation capabilities

To assess the growth and degradation capabilities of the FD, TD, and MD consortia, inocula (5%) were introduced into Erlenmeyer flasks containing MSM medium amended with FIP, THIO, and the FIP + THIO mixture at a concentration of 50 μg/mL. These experiments were performed in triplicate, with additional triplicate flasks serving as controls with no consortia inoculation. After 10 days of incubation (30°C, 120 rpm, in darkness), samples were periodically taken from each flask to evaluate pesticide degradation and microbial growth. In parallel experiments, employing the same incubation conditions and operational procedures, the selected consortia’s capability to degrade varying concentrations of FIP and THIO (25, 50, 100, 200, 400, 600, and 800 μg/mL), either alone or in combination with one another, was also investigated.

### 2.4 Optimization of FIP and THIO degradation conditions with the consortia

To assess the impact of various factors on the degradation of FIP and THIO, the response surface methodology (RSM) with a Box–Behnken design was employed (Yang et al., 2018; Zhan et al., 2018; Pang et al., 2023). The Box–Behnken design was structured with three key variables: pH (5, 7, and 10), pesticide concentration (25, 50, and 100 μg/L), and inoculum size (1, 5, and 10%). This design encompassed 17 distinct experiments conducted in triplicates to ensure reliability and precision (Supplementary Table S4). The percentage of degradation achieved for FIP and THIO, either alone or as a mixture, was chosen as the dependent variable for the analysis.

### 2.5 Assessment of consortia performance in paddy soils

The proficiency of the FD, TD, and MD consortia in pesticide degradation was examined within paddy soils devoid of any prior exposure to FIP or THIO for a minimum of 2 years. These investigations were carried out in microcosms comprising 100 mL amber glass vials, each containing 40 g of either sterile or non-sterile air-dried soil. Aqueous solutions of FIP, THIO, and the FIP + THIO mixture were prepared by introducing stock solutions of each pesticide in Ace into deionized water. After 24 h of Ace evaporation, solutions were filter-sterilized and utilized to spike soil samples at 15 and 150 μg/g while adjusting the soil moisture to 20 and 100% (*v/w*). The microcosms were then categorized into two groups. The first group received inocula of the FD, TD, and MD consortia (5%), each added to the glasses spiked with FIP, THIO, and the FIP + THIO mixture, respectively. The second group, however, remained uninoculated, serving as the control. A summary of soil experimental design is presented in Supplementary Table S5. A total of 24 experiments in triplicate consisting of 72 microcosms were prepared. Soil microcosms were incubated in darkness at 30°C for 14 days. At predetermined intervals, subsamples were collected and subjected to HPLC analysis to measure the decline in THIO and FIP residues.

### 2.6 Identification of bacterial consortia

The QIAamp DNA Stool Mini Kit (Qiagen, Germany) was employed for DNA extraction from the FD, TD, and MD consortia, following the manufacturer’s instructions. Subsequent PCR amplification and sequencing procedures were performed at Beijing Novogene Bioinformatic Technology Co., Ltd. (Tianjin, China). To target the V3-V4 region of the 16S rRNA gene, specific primers 341F-806F with a barcode (341F: CCTAYGGGRBGCASCAG and 806R: GGACTACNNGGGTATCTAAT) were utilized for PCR amplification (Niem et al., 2020). The PCR program (T100, Bio-Rad, United States) consisted of an initial denaturation at 98°C for 1 min, followed by 30 cycles including denaturation at 98°C for 10 s, annealing at 50°C for 30 s, and extension at 72°C for 30 s. The final extension step was conducted at 72°C for 5 min. Subsequently, PCR products underwent purification and recovery using the GeneJET Gel Extraction Kit (Thermo Scientific, United States). Library preparation was accomplished using the TruSeq DNA PCR-Free Library Preparation Kit (Illumina, United States), with quantification and detection performed using the Qubit system (Thermo Scientific, United States). After successfully passing the quality assessment, high-throughput sequencing was executed on the Illumina NovaSeq 6,000 platform.

Raw sequences were imported into QIIME2-2023.5 and analyzed within QIIME 2 (Bolyen et al., 2019). Demultiplexing, adapter sequence, and primer trimming were conducted using the cutadapt plugin (Martin, 2011). Denoising, quality and chimera filtering, and dereplication of amplicon sequence variants (ASVs) were performed utilizing DADA2 (Callahan et al., 2016). Taxonomic assignment of the obtained ASVs was executed using the classify-sklearn method with a Naive Bayes supervised learning algorithm. This algorithm was based on a trained classifier using Greengenes 13_5 v references (Lin and Ju, 2023) with a 97% similarity threshold. Alpha diversity metrics, including Shannon and Faith’s phylogenetic diversity (Faith’s_PD) indexes, were computed using QIIME2. The pairwise Kruskal–Wallis test was applied to assess significant differences (Lozupone et al., 2006). Finally, a phylogenetic tree was constructed using the impress plugin from QIIME2. Raw sequences can be found in the sequence read archive (SRA) of the NCBI with the BioProject accession number PRJNA1061744.

### 2.7 Analytical methods

To evaluate the dissipation of FIP and THIO residues in both MSM cultures and soils, the extraction method based on the original unbuffered QuEChERS approach proposed by Anastassiades et al. (2003) was followed. In total, 5 g of samples was extracted using 10 mL of MeCN. Subsequently, a salting-out step involved the addition of 4 g MgSO_4_ and 1 g NaCl to each sample. After thorough mixing and centrifugation at 5,000 rpm for 5 min, 2 mL of the resulting supernatant was subjected to a dispersive solid-phase extraction (d-SPE) cleanup step, utilizing 300 mg MgSO_4_ and 50 mg PSA. After being vigorously shaken for 1 min and centrifuged at 5,000 rpm for 4 min, the supernatant was evaporated to dryness using N_2_ and then reconstituted in 250 μL MeOH for subsequent HPLC analysis. For the quantification of FIP and THIO, HPLC with a UV/VIS detector was employed (Shimadzu, LC9A). Specific wavelengths of 280 and 233 nm were selected for FIP and THIO, respectively. Separation was achieved using a C_18_ column (150 × 4.6 mm, 5 μm) at a temperature of 40°C, with a mobile phase consisting of a 70:30 (*v/v*) mixture of MeCN and water in isocratic elution mode, with a flow rate of 1 mL/min.

A comprehensive set of method validation procedures was conducted following SANTE guidelines (SANTE, 2021), to validate the extraction and analysis methods. Precision and accuracy were assessed by calculating the recoveries and relative standard deviations (RSDs) of FIP and THIO spiked into pesticide-free soils or MSM cultures. The instrumental detection and quantification limits (IDL and IQL), as well as the estimated method detection and quantification limits (EMDL and EMQL), were determined using the slope (m) and root mean square error (RMSE) of the solvent calibration curve in MeOH. Linearity was assessed through matrix-matched calibration curves for each pesticide, ranging from EMQL to 10 EMQL (see Supplementary material and Supplementary Table S6 for detailed information on method validation protocols and results).

To extract metabolites produced during FIP and THIO degradation in MSM cultures, the same procedure as described for the parent compounds was followed. However, during the final evaporation stage, the residue was dissolved in 250 μL MeCN. To detect the metabolites, gas chromatography–mass spectrometry (GC–MS) was used, with an Agilent 6,890 N GC equipped with an Agilent 5,973 N MS detector featuring an electron ionization source (electron energy: 70 eV, solvent delay: 60 min, and mass range: 30–250 m/z) (Agilent Technologies, United States). Separations were conducted using an HP-5 ms column (30 m × 0.25 mm × 0.25 μm) with helium (purity >99.999%) as the carrier gas. The detector, ion source, MS transmission line, and quadrupole temperatures were set to 320, 230, 280, and 150°C, respectively.

For FIP metabolites, the carrier gas flow was set at 1.5 mL/min, with an injector temperature of 280°C in splitless mode and an injection volume of 2 μL. The oven temperature program was as follows: the initial temperature was set at 100°C for 1 min, then raised to 250°C at a rate of 10°C/min for 2 min, and finally increased to 280°C at a rate of 10°C/min for 5 min. For THIO metabolites, the carrier gas was set at 1 mL/min, with the injector temperature of 280°C in split mode (split ratio: 1:10). The oven temperature program was as follows: the initial temperature was set at 50°C for 30 s, then increased to 190°C at a rate of 10°C/min for 1 min, and finally raised to 280°C at 10°C/min for 2 min.

### 2.8 Statistical analyses

The degradation rate (k) and the half-life (t_1/2_) for FIP and THIO in the culture medium and soil were assessed by modeling the data with the first-order exponential equation (Eqs. 1, 2) using TableCurve 2D v5.0 (Torabi et al., 2017):


(1) $$C_{t}=C_{0}\times e^{-kt}$$

(2) $$t_{1/2}=\ln\left(2\right)/k$$

where C_0_ denotes the initial concentration of either FIP or THIO (μg/ml); k represents the degradation rate (days^−1^); t signifies the degradation time (days); and C_t_ denotes the concentration of FIP or THIO at a specific time, t (μg/ml).

Degradation kinetic parameters of FIP, THIO, and FIP + THIO mixture were assessed across various initial concentrations with the FD, TD, and MD consortia, respectively, using the Andrews equation (Eq. 3) in TableCurve 2D v5.0 (Chen et al., 2013; Yang et al., 2018):


(3) q=qmaxCC+Ks+C2Ki

In this equation, C corresponds to the concentration of FIP or THIO (μg/ml), q represents the specific degradation rate of FIP or THIO (day^−1^), q_max_ signifies the maximum specific degradation rate of FIP or THIO (day^−1^), K_s_ is the half-saturation constant (μg/ml), and K_i_ stands for the inhibition constant for FIP or THIO (μg/ml).

To optimize the degradation of FIP and THIO by the selected consortia, RSM regression (Eq. 4) was employed using R software version 4.3.1.:


(4) $$Y_{i}=b_{0}+\sum b_{i}X_{i}+\sum b_{ij}X_{i}X_{j}+\sum b_{ii}X_{i}^{2}$$

Here, Y_i_ is the predicted response, X_i_ and X_j_ are the variables, and b_0_, b_i_, b_ij_, and b_ii_ represent the coefficients of the respective terms. Subsequently, this is allowed to plot a response surface, following the study by Pang et al. (2023).

In the context of soil experiments, analysis of variance (ANOVA) in combination with Tukey’s honestly significant difference (Tukey’s HSD) post-hoc test was utilized to compare degradation percentages between different treatments. This analysis was performed using R software version 4.3.1. Furthermore, the contribution of the addition of consortium to the degradation rates of pesticides (k_+/−_) was assessed by measuring the k ratios in soils with and without inoculation, as specified in Eq. 5:


(5) k+−=kinoculatedsoil/kuninoculatedsoil

Principal coordinate analysis (PCoA) of consortia bacterial communities was conducted in R software version 4.3.1 using the Hellinger transformation.

## 3 Results

### 3.1 Characterization of FD, TD, and MD consortia

**Figure 1:**
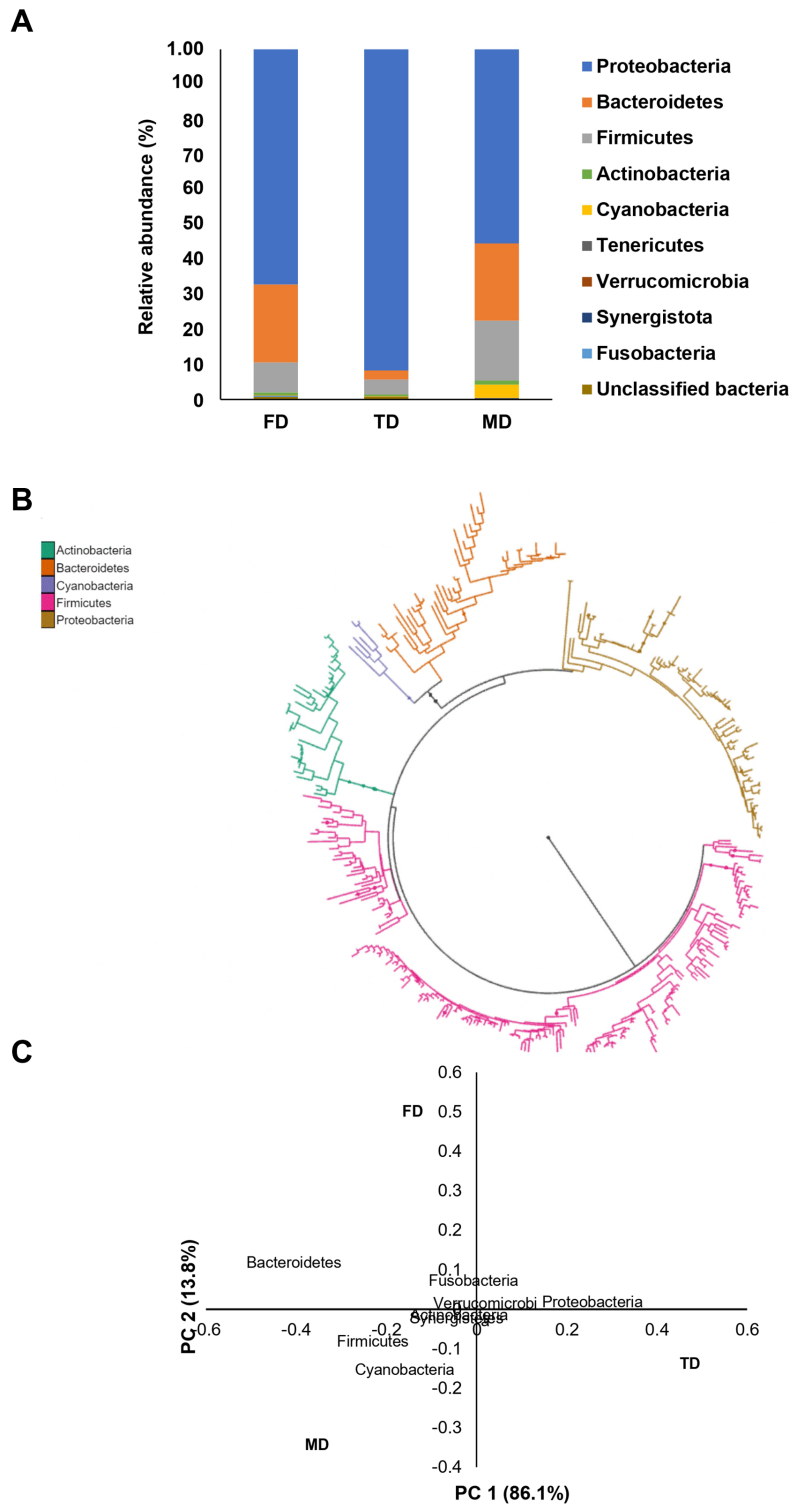
**(A)** Relative abundance of the bacteria present in the isolated consortia at the phylum level, **(B)** phylogenetic tree based on the most abundant bacterial phyla in the three isolated consortia, and **(C)** PCoA plot of the isolated bacterial consortia.

**Figure 2:**
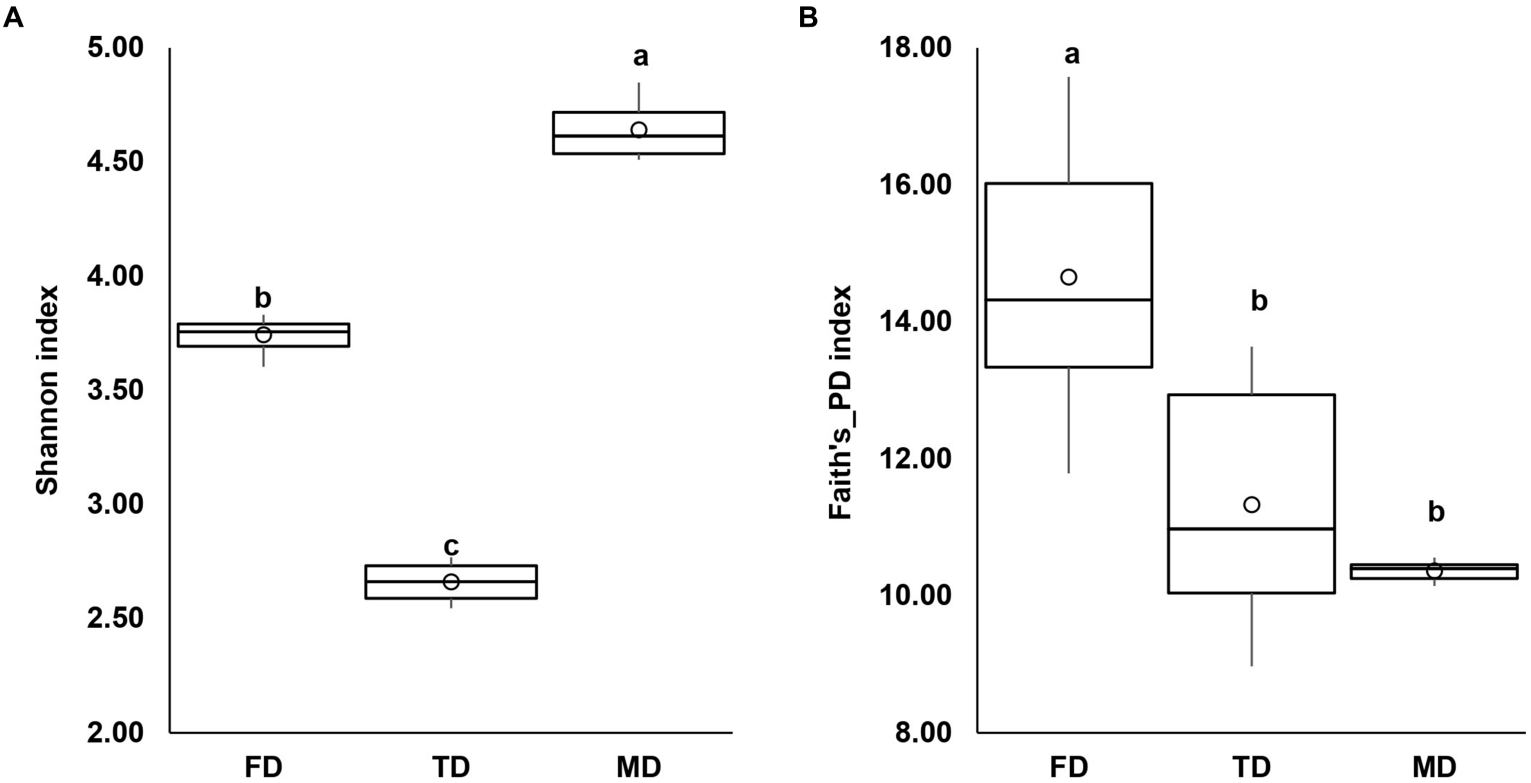
Box plots showing Shannon diversity **(A)** and Faith’s_PD **(B)** indexes for the isolated consortia. Different letters show significant differences (Kruskal–Wallis test, *p* < 0.05).

In this investigation, three bacterial consortia capable of degrading FIP, THIO, and the FIP + THIO mixture were isolated and designated as FD, TD, and MD, respectively. The FD consortium showcased a diverse composition, encompassing 102 genera, 61 families, 31 orders, 19 classes, and 10 phyla. The TD consortium comprised 95 genera, 64 families, 30 orders, 20 classes, and 9 phyla. Meanwhile, the MD consortium consisted of 41 genera, 35 families, 17 orders, 11 classes, and 7 phyla (Figure 1). Alpha diversity indices were calculated for each consortium. The MD consortium exhibited the highest Shannon diversity (*p* < 0.01), followed by FD and TD consortia (Figure 2A). In terms of Faith’s_PD, the FD consortium had the highest value (*p* < 0.01), while MD and TD had lower Faith’s_PD values, with no significant difference between them (*p* > 0.05) (Figure 2B).

Proteobacteria dominated all consortia (FD: 67%, TD: 92%, and MD: 72%), with *Azospirillum* sp. as the most abundant genus (FD: 69%, TD: 87%, and MD: 48%). An unclassified genus of Rhizobioales was significant in FD (26%) and MD (12%). Notably, in MD, *Ochrobactrum* sp., *Shinella* sp., *Sphingobium* sp., and *Sphingomonas* sp. were present at relative abundances of 17, 7, 4, and 3%, respectively (Figure 1; Supplementary Figure S1). Bacteroidetes constituted the second-highest phylum in FD and MD (22%), contrasting with only 3% in TD. *Chitinophaga* sp. dominated Bacteroidetes in FD (62%) and MD (56%), followed by an unclassified Bacteroidales genus (30 and 34%, respectively) and *Bacteroides* sp. (5 and 6%, respectively) (Figure 1; Supplementary Figure S2). Firmicues represented 9, 4, and 17% of FD, TD, and MD consortia, respectively. *Oscillospira* sp. and *Dorea* sp. and genera of Ruminococcaceae, Lachnospiraceae, and Clostridiales were most abundant (Figure 1; Supplementary Figure S3). Actinobacteria constituted 1% of each consortium. *Bifidobacterium* sp., *Adlercreutzia* sp., *Mycobacterium* sp., and *Curtobacterium* sp. and unclassified genera in Coriobacteriaceae and Bifidobacteriaceae comprised over 90 and 70% of bacteria in FD and TD, respectively. In MD, *Bifidobacterium* sp. (55%) and *Acinetobacter* sp. (33%) were predominant (Figure 1; Supplementary Figure S4).

PCoA accounted for 99% of the bacterial variation at the phylum level. The phyla with the longest negative loadings on PC1 (Cyanobacteria, Firmicutes, and Bacteroidetes) influenced the scores mostly of consortia MD and FD with their spatial, whereas the variables with positive loadings (Proteobacteria) influenced mainly the scores of TD consortium. The phyla with loadings of positive signs in PC2 (Fusobacteria, Bacteroidetes) influenced consortium FD while phyla with loadings of negative signs in PC2 (Firmicutes, Cyanobacteria, Actinobacteria, Synergistetes, and Verrucomirobia) influenced consortia TD and MD (Figure 1C).

### 3.2 Growth and degradation efficiencies of FD, TD, and MD consortia

**Figure 3:**
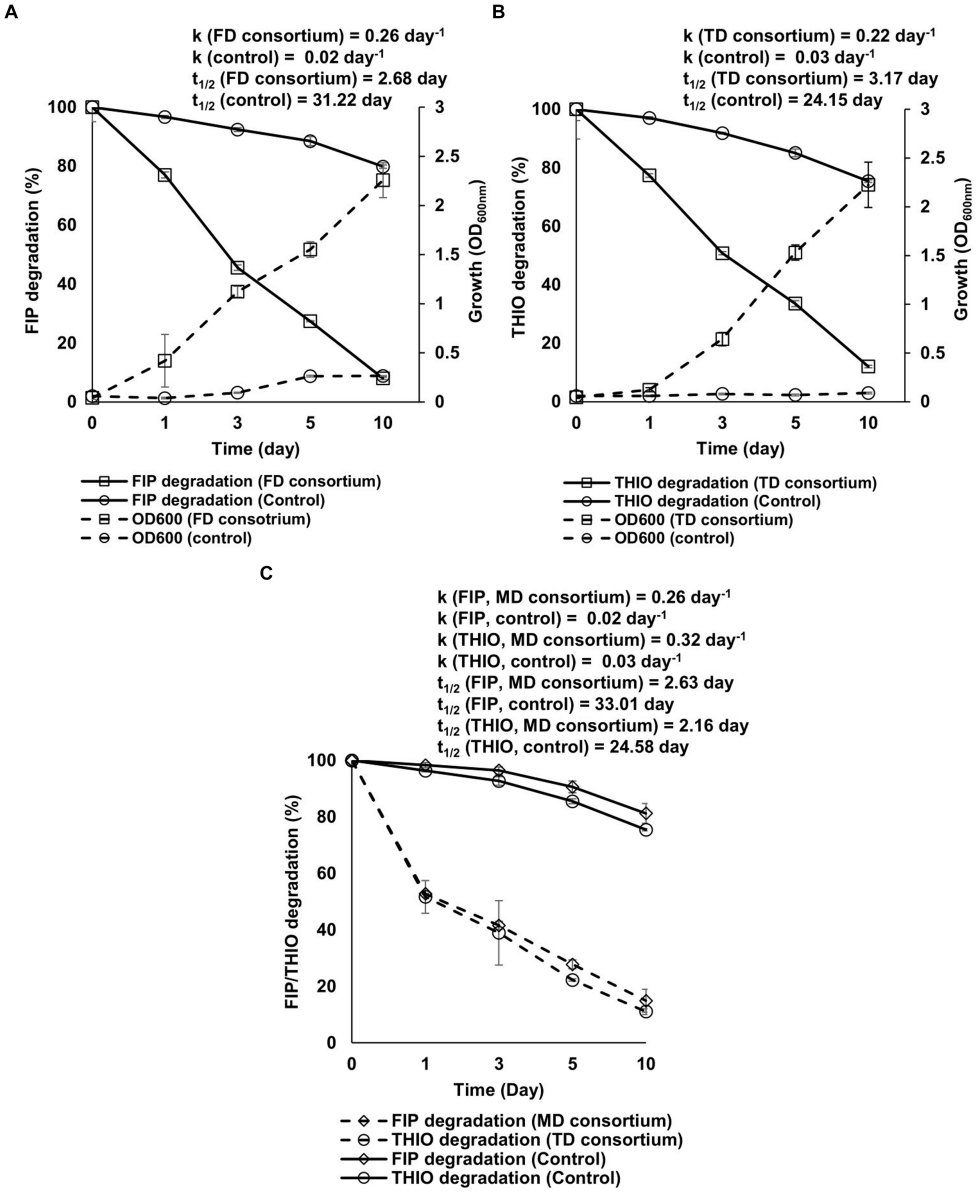
Growth-linked degradation of **(A)** FIP, **(B)** THIO, and **(C)** FIP + THIO mixture in MSM inoculated with the FD, TD, and MD consortia, respectively. Kinetic parameters derived from the first-order exponential equation (Eqs. 1, 2) are reported. In the case of the FIP and THIO mixture, the initial turbidity of the culture, resulting from adding two pesticide solutions, prevented the measurement of OD_600_. Error bars represent standard deviations (*n* = 3).

The results indicate that all three consortia possess robust growth and effective degradation of FIP, THIO, and the FIP + THIO mixture in the MSM cultures within 10 days after incubation (Figure 3). Specifically, the FD and TD consortia exhibited notable 9- and 25-fold increases in culture OD_600_, respectively, compared with the control (Figures 3A,B). In the presence of FD and TD consortia, the degradation rates of FIP and THIO were accelerated by 13 and 7 times, respectively, compared with uninoculated cultures (Figures 3A,B). Similarly, the MD consortia significantly enhanced the degradation rates of FIP and THIO, showing 13- and 11-fold increases compared with the control (Figure 3C).

**Figure 4:**
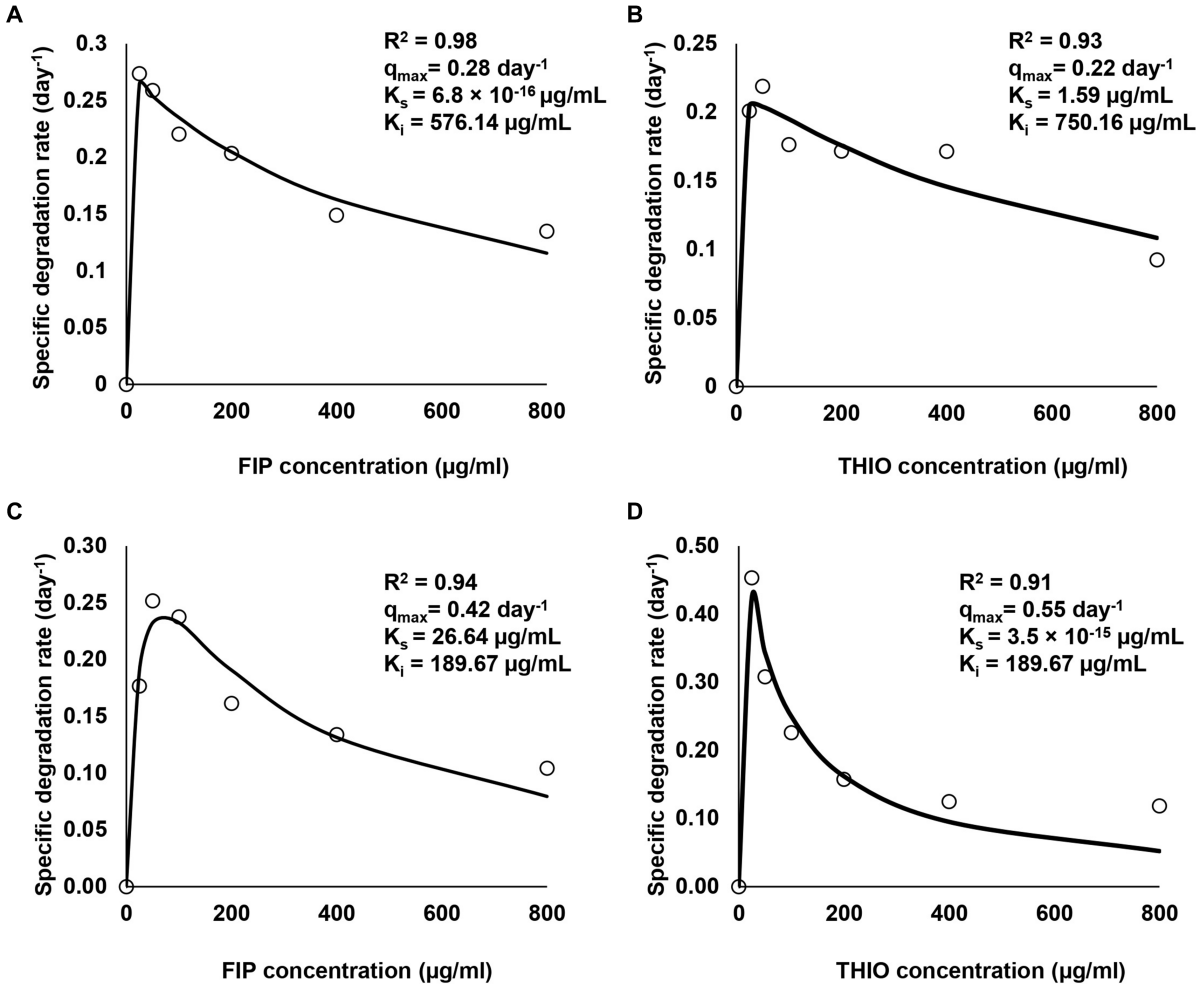
Relationship between initial concentrations of FIP and THIO and their specific degradation rate by FD, TD, and MD consortia. **(A,C)** Degradation of FIP concentrations by FD and MD consortia, respectively, **(B,D)** degradation of THIO concentrations by TD and MD consortia, respectively.

The degradation of various concentrations of FIP and THIO by three consortia, along with associated kinetic parameters such as R^2^, q_max_, K_s_, and K_i_, is shown in Figure 4. The findings indicate that all consortia exhibited tolerance to elevated levels of FIP and THIO, successfully degrading these pesticides within the concentration range of 25–800 μg/mL. However, as the pesticide concentration increased, the degradation efficiency of the consortia decreased. Specifically, for FIP, the FD and MD consortia achieved the highest degradation rates at 25 and 50 μg/mL, registering 0.25 and 0.23 day^−1^, respectively. Nevertheless, at concentrations exceeding 50 μg/mL, a decline in FIP degradation rates was observed, reaching the lowest values at 800 μg/mL for the FD and MD consortia (0.12 and 0.08 day^−1^, respectively), indicating an inhibitory effect of high FIP concentrations on the consortia (Figures 4A,C).

Similar trends were observed for THIO, with the TD and MD consortia exhibiting the highest degradation rates (0.20 and 0.42 day^−1^) at 50 and 25 μg/mL, respectively. Conversely, the inhibition of THIO dissipation rates was noted at 800 μg/mL with the TD and MD consortia (0.11 and 0.05 day^−1^, respectively) (Figures 4B,D).

### 3.3 Optimization of FIP and THIO degradation with FD, TD, and MD consortia

The optimization of FIP and THIO degradation conditions was conducted through an RSM regression, employing a Box–Benken design with 17 experiments (Supplementary Table S4). Three crucial independent variables, such as culture pH (X_1_), pesticide concentration (X_2_), and inoculum size (X^3^), were selected for this study. The results revealed that the measured degradation of FIP ranged from 39.5 to 96.4% with the FD consortium and from 5.1 to 89.1% with the MD consortium. Similarly, for THIO, measured degradation percentages varied between 1.7 and 87.7% with the TD consortium and between 2.0 and 85.9% with the MD consortium (Supplementary Table S4). Quadratic polynomial models were employed to fit the degradation data for FIP by the FD and MD consortia (Eqs. 6, 7, respectively) and for THIO by the TD and MD consortia (Eqs. 8, 9, respectively).


(6) $$\begin{array}{l} Y_{i}=-191.4+65.93X_{1}+0.905X_{2}-1.703X_{3}+0.01645X_{1}X_{2}\\ \;+0.5121X_{1}X_{3}+0.0008305X_{2}X_{3}-4.656X_{1}^{2}\\ \;-0.005367X_{2}^{2}-0.3126X_{3}^{2} \end{array}$$

(7) $$\begin{array}{l} Y_{i}=-360.7+101X_{1}+0.4666X_{2}+18.71X_{3}+0.1296X_{1}X_{2}\\ \;-0.5111X_{1}X_{3}-0.05393X_{2}X_{3}-7.009X_{1}^{2}\\ \;-0.00793X_{2}^{2}-1.031X_{3}^{2} \end{array}$$

(8) $$\begin{array}{l} Y_{i}=-421.74905+106.64224X_{1}+3.02409X_{2}+9.82383X_{3}\\ \;-0.15639X_{1}X_{2}+0.28766X_{1}X_{3}-0.03771X_{2}X_{3}-6.86698X_{1}^{2}\\ \;-0.01036X_{2}^{2}-1.05981X_{3}^{2} \end{array}$$

(9) $$\begin{array}{l} Y_{i}=-503+132X_{1}+1.501X_{2}+13.16X_{3}+0.05341X_{1}X_{2}\\ \;-0.4438X_{1}X_{3}+0.02262X_{2}X_{3}-8.665X_{1}^{2}\\ \;-0.0148X_{2}^{2}-0.9755X_{3}^{2} \end{array}$$

Table 1 provides a comprehensive analysis of variance (ANOVA) results for FIP and THIO. The high determination coefficient (R^2^ > 0.9), accompanied by significant model *F* values (*p* < 0.01) and non-significant lack-of-fit F values (*p* > 0.05), attests to the robustness of our predictive models for FIP and THIO degradation by the consortia. The influence of pH, pesticide concentration, and inoculum size on FIP and THIO dissipation was found to be statistically significant with the FD and TD consortia, respectively (*p* < 0.01). Interestingly, for the MD consortium, only pesticide concentration exhibited a significant effect on THIO degradation (*p* = 0.01) (Table 1). In contrast, for the MD consortia, interaction effects and quadratic terms of each factor significantly impacted the degradation of both FIP and THIO (*p* < 0.05).

**Table 1:** Analysis of variance for the quadratic FIP and THIO degradation models by the selected consortia.

Source	df	FD consortium				TD consortium				MD consortium							
		FIP				THIO				FIP				THIO			
		SS	MS	*F*-value	*p*-value	SS	MS	F-value	*p*-value	SS	MS	F-value	*p*-value	SS	MS	F-value	*p*-value
Model	9	16802.02	1866.89	99.96	< 0.01	44667.44	4963.05	115.6	< 0.01	34478.48	3830.94	31.89	< 0.01	46817.15	5201.91	341.5	< 0.01
$X_{1}$	1	334.18	334.18	17.89	< 0.01	4502.34	4502.34	73.70	< 0.01	285.17	285.17	3.39	0.07	26.52	26.52	1.60	0.21
$X_{2}$	1	3641.24	3641.24	194.97	< 0.01	3768.45	3768.45	61.69	< 0.01	150.69	150.69	1.79	0.19	121.14	121.14	7.31	0.01
$X_{3}$	1	1084.16	1084.16	58.05	< 0.01	2316.50	2316.50	37.92	< 0.01	50.62	50.62	0.60	0.44	14.86	14.86	0.90	0.35
$X_{1}X_{2}$	1	26.83	26.83	1.44	0.24	2770.72	2770.72	45.36	< 0.01	1966.62	1966.62	23.38	< 0.01	329.68	329.68	19.91	< 0.01
$X_{1}X_{3}$	1	407.92	407.92	21.84	< 0.01	146.03	146.03	2.39	0.13	365.98	365.98	4.35	0.04	322.45	322.45	19.47	< 0.01
$X_{2}X_{3}$	1	0.25	0.25	0.01	0.91	515.22	515.22	8.43	0.01	1054.20	1054.20	12.53	< 0.01	185.50	185.50	11.20	< 0.01
$X_{1}^{2}$	1	10205.2	10205.2	546.44	< 0.01	22899.86	22899.86	374.87	< 0.01	23605.10	23605.10	280.66	< 0.01	36422.84	36422.84	2199.23	< 0.01
$X_{2}^{2}$	1	603.52	603.52	32.32	< 0.01	2366.84	2366.84	38.75	< 0.01	1453.97	1453.97	17.29	< 0.01	4534.61	4534.61	273.80	< 0.01
$X_{3}^{2}$	1	498.72	498.72	26.70	< 0.01	5381.49	5381.49	88.10	< 0.01	5546.11	5546.11	65.94	< 0.01	4859.55	4859.55	293.42	< 0.01
Residual	41	765.70	18.68	-	-	2504.58	61.09	-	-	3448.36	84.11	-	-	679.03	16.56	-	-
Lack of Fit	17	85.07	5.01	0.18	0.99	278.28	16.37	0.18	0.99	383.15	22.54	0.18	0.99	75.45	4.44	0.18	0.99
Pure error	24	680.66	28.36	-	-	2226.29	92.76	-	-	3065.21	121.72	-	-	603.58	25.15	-	-
Total	50	17567.73	351.35	-	-	47172.01	943.44	-	-	37926.84	758.53	-	-	47496.18	949.92	-	-
Adjusted R-squared:		0.95				0.94				0.91				0.98			

X_1_: Culture pH, X_2_: pesticide concentration (μg/ml), X_3_: Inoculum size (%).

**Figure 5:**
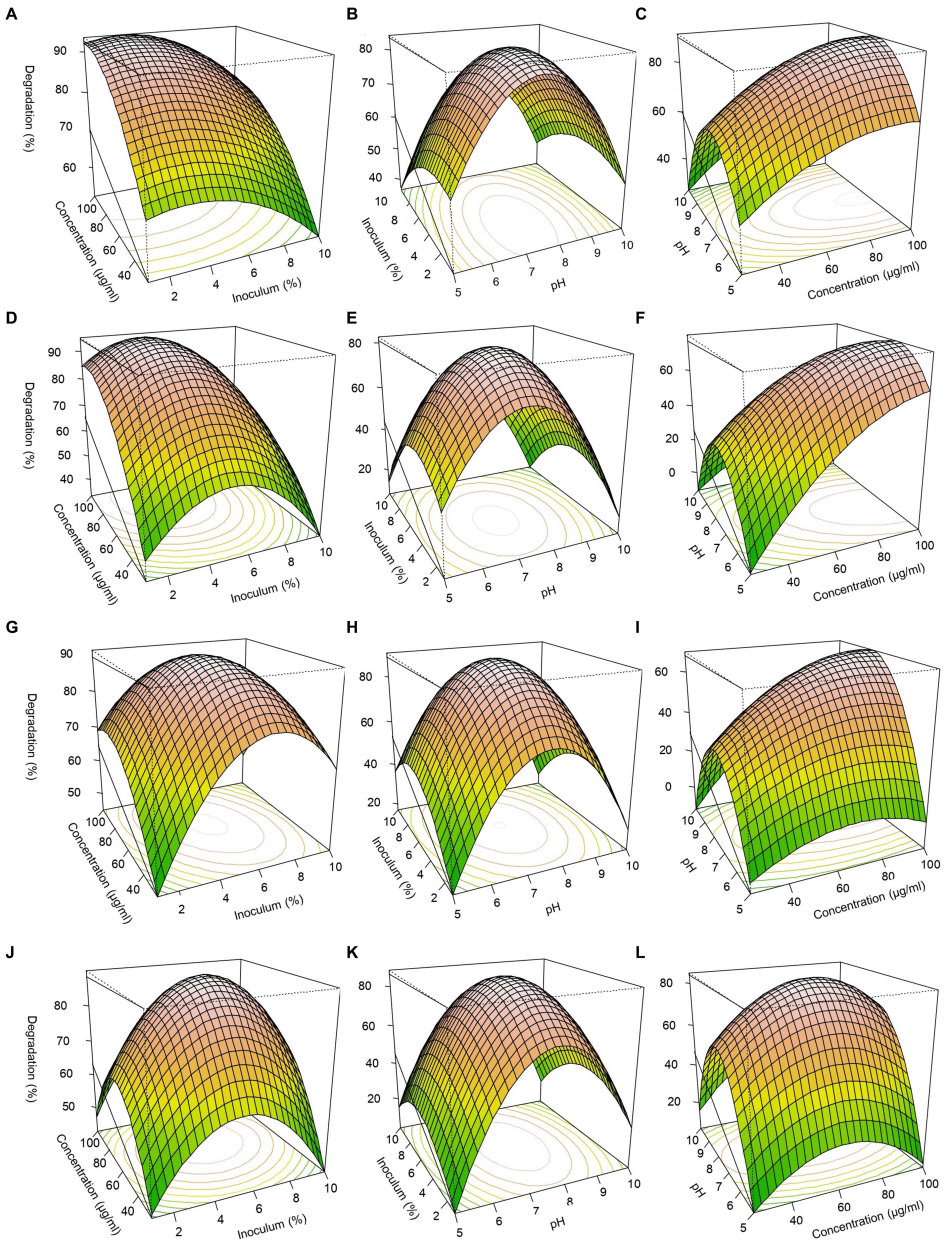
Response surface 3D graphs for FIP and THIO degradation optimization by FD, TD, and MD consortia. **(A,G)** Effect of pesticide concentrations and inoculum size on FIP degradation by FD and MD consortia, respectively, **(D,J)** effect of pesticide concentrations and inoculum size on THIO degradation by TD and MD consortia, respectively, **(B,H)** effect of pH and inoculum size on FIP degradation by FD and MD consortia, respectively, **(E,K)** effect of pH and inoculum on THIO degradation by TD and MD consortia, respectively, **(C,I)** effect of pesticide concentrations and pH on FIP degradation by FD and MD consortia, respectively, **(F,L)** effect of pesticide concentrations and pH on THIO degradation by TD and MD consortia, respectively.

Three-dimensional response surfaces were constructed with pH, pesticide concentration, and inoculum size (Figure 5). For FIP degradation, the optimal conditions with FD and MD consortia, achieving approximately 94% predicted degradation, were identified at pH 7.5–7.7, a pesticide concentration of 70–100 μg/mL, and an inoculum size of 4–6% (Figures 5A–C,G). Similarly, for THIO degradation, optimal conditions resulting in approximately 94% predicted degradation were determined at pH 7–7.7, a pesticide concentration of 68–70 μg/mL, and an inoculum size of 4–6% (Figures 5D–F,I).

### 3.4 Metabolic pathway of FIP and THIO degradation with FD, TD, and MD consortia

**Figure 6:**
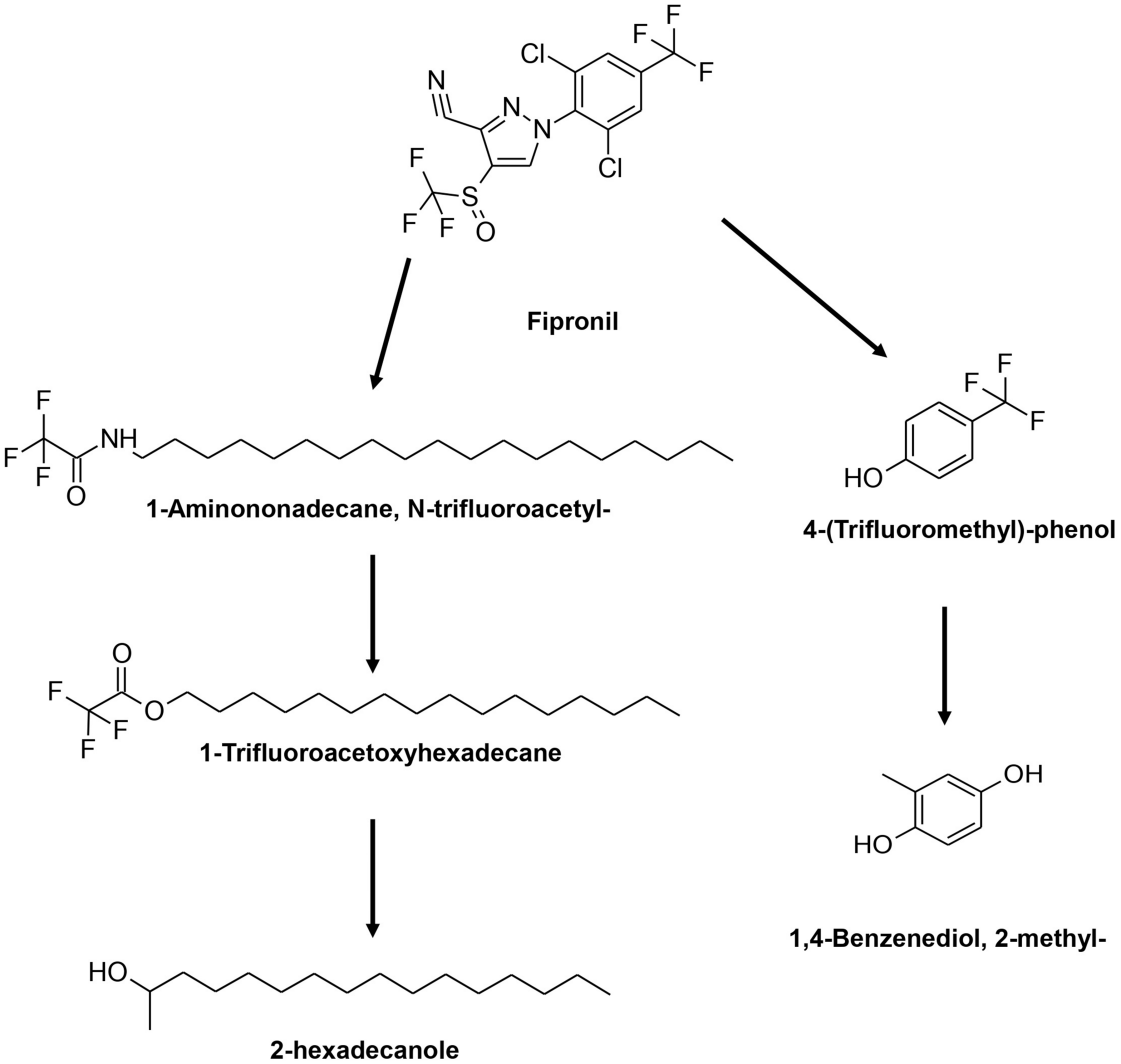
Proposed degradation pathway for FIP by FD and MD consortia.

GC–MS results demonstrated comparable metabolite profiles in the degradation of FIP by the FD and MD consortia (Table 2; Supplementary Figure S5). Analysis of these metabolites facilitated the formulation of a novel pathway for FIP degradation by the FD and MD consortia (Figure 6). According to this proposed pathway, FIP initially underwent a hydrolytic reaction, yielding 1-aminononadecane, N-trifluoroacetyl, and 4-(trifluoromethyl)-phenol. Subsequently, these metabolites transformed into 1-trifluoroacetoxyhexadecane, 2-hexadecanole, and 1,4-benzenediol through oxidative or hydrolytic processes.

**Table 2:** Intermediate transformation products during FIP degradation by FD and MD consortia.

Compound name	RT (min)	MW (g/mol)	Formula
1-Trifluoroacetoxyhexadecane	11.08	338.4	C_18_H_33_F_3_O_2_
2-hexadecanole	13.66	242.44	C_16_H_34_O
1-Aminononadecane, N-trifluoroacetyl-	14.70	379.5	C_21_H_40_F_3_NO
1,4-Benzenediol, 2-methyl-	21.26	124.14	C_7_H_8_O_2_
4-(Trifluoromethyl)-phenol	25.34	1692.11	C_7_H_5_F_3_O

RT: Retention time, MW: molecular weight.

**Figure 7:**
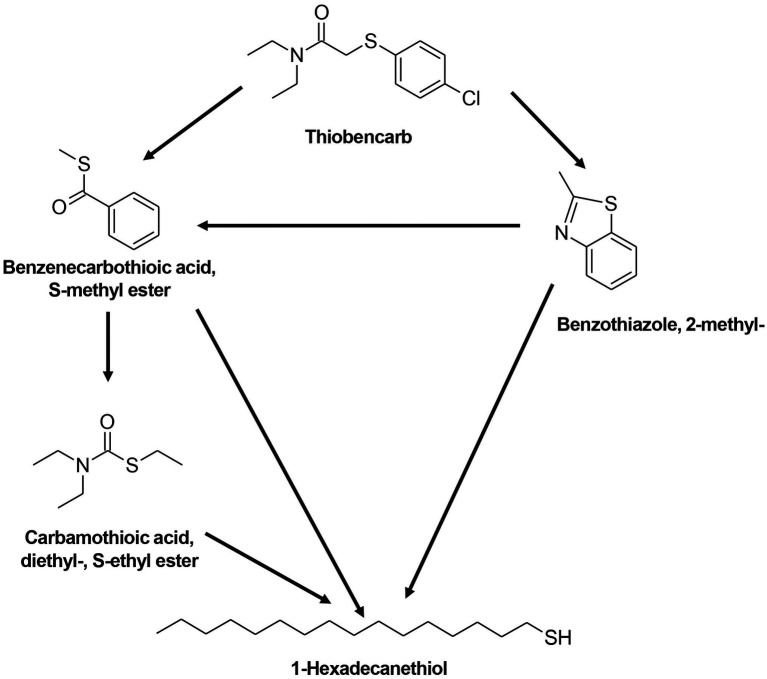
Proposed degradation pathway for THIO by TD and MD consortia.

Similar to FIP, THIO degradation with the TD and MD consortia also resulted in comparable metabolites (Table 3; Supplementary Figure S6). The proposed pathway for THIO degradation indicates the cleavage of the C-S bond, leading to the production of benzenecarbothioic acid, S-methyl ester, and subsequently carbamothioic acid, diethyl-, and S-ethyl ester (Figure 7). Additionally, benzothiazole and 2-methyl-1-hexadecanethiol were observed as products of THIO degradation with the TD and MD consortia.

**Table 3:** Intermediate transformation products during THIO degradation by TD and MD consortia.

Compound name	RT (min)	MW (g/mol)	Formula
Benzothiazole, 2-methyl-	13.803	149.21	C_8_H_7_NS
1-Hexadecanethiol	18.63	258.51	C_16_H_34_S
Carbamothioic acid, diethyl-, S-ethyl ester	20.77	161.27	C_7_H_15_NOS
Benzenecarbothioic acid, S-methyl ester	22.103	152.21	C_8_H_8_OS

RT: Retention time, MW: molecular weight.

### 3.5 FIP and THIO degradation in soil with FD, TD, and MD consortia

Significant improvements in FIP degradation (*p* < 0.01) were evident in both sterile and non-sterile soils at 20 and 100% moisture conditions, following the inoculation of FD and MD consortia (Tables 4, 5). In non-inoculated soils, the half-life (t_1/2_) of FIP in sterile and non-sterile samples ranged from 14 to 139 and 8 to 27 days, respectively. Upon inoculation with FD or MD consortia, the t_1/2_ of FIP decreased to 8–18 and 4–20 days in sterile and non-sterile soils, respectively (Tables 4, 5). It is particularly noteworthy that FD’s more pronounced effect on sterile soils at 20% moisture result in a significant increase in FIP degradation (*p* < 0.01) and k_(+/−)_ values of 6.80 and 9.53 at 15 and 150 μg/g, respectively (Table 4). Under 100% moisture conditions, FD consortia were significantly effective (*p* < 0.01), although the effect was less pronounced compared with 20% moisture, yielding k_(+/−)_ values of 1.82 and 3.94 at 15 and 150 μg/g, respectively (Table 4). Similarly, MD consortia exhibited significant efficacy in FIP degradation at 20% moisture in non-sterile and sterile soils compared with 100% moisture (*p* < 0.01). However, at 15 μg/g, the effect of MD consortia was not significant at 100% moisture content (*p* > 0.05) (Table 5).

**Table 4:** Kinetics for FIP degradation in soil by the FD consortium.

Pesticide concentration (μg/g)	Soil moisture (%)	Soil sterility	Consortium inoculation^*^	D (%) ± SD^**^	t_1/2_ (day)^***^	R^2****^	k^*****^ ± SE (day^−1^)	k^******^_(+/−)_ ± SE
15	100	Sterile	+	67.69^bc^ ± 1.31	9	0.91	0.080 ± 0.007	1.82 ± 0.18
			−	48.56^fg^ ± 2.06	16	0.97	0.044 ± 0.002	
		Non-sterile	+	78.56^a^ ± 0.46	7	0.99	0.106 ± 0.004	1.29 ± 0.06
			−	68.80b ± 2.57	8	0.99	0.082 ± 0.003	
	20	Sterile	+	43.26^gh^ ± 1.24	18	0.90	0.039 ± 0.004	6.80 ± 1.01
			−	7.78^j^ ± 1.48	121	0.90	0.006 ± 0.001	
		Non-sterile	+	55.00^def^ ± 3.35	13	0.94	0.053 ± 0.004	1.62 ± 0.14
			−	36.88^h^ ± 2.95	21	0.96	0.033 ± 0.002	
150	100	Sterile	+	52.14^ef^ ± 0.93	13	0.90	0.054 ± 0.007	3.94 ± 0.57
			−	18.38^i^ ± 0.96	51	0.92	0.014 ± 0.001	
		Non-sterile	+	73.58^ab^ ± 1.33	7	0.99	0.093 ± 0.002	1.30 ± 0.06
			−	60.64^cd^ ± 2.82	10	0.97	0.072 ± 0.003	
	20	Sterile	+	59.48^de^ ± 3.54	11	0.98	0.065 ± 0.002	9.53 ± 0.90
			−	9.24^j^ ± 1.46	102	0.91	0.007 ± 0.001	
		Non-sterile	+	37.61^h^ ± 0.35	20	0.98	0.034 ± 0.001	1.06 ± 0.05
			−	36.70^h^ ± 0.71	21	0.98	0.032 ± 0.001	

SE: Standard error (*n* = 3). SD: Standard deviation (*n* = 3). ^*^ + and – indicate soils with and without consortium inoculation, respectively. ^**^Degradation after 14 days. Values with the same lowercase superscript letters in the column do not defer significantly (Tukey’s HSD post-hoc test, *p* = 0.05). ^***^Half-lives of FIP are calculated based on Eq. 2. ^****^The first-order exponential decay model (Eq. 1) coefficient of determination. ^*****^FIP degradation rates based on the first-order exponential decay model (Eq. 1). ^******^The contribution of the FD consortium to the degradation rates of FIP (Eq. 5). Standard errors are calculated via the error propagation method.

**Table 5:** Kinetics for FIP and THIO degradation in soil by the MD consortium.

Pesticide	Pesticide concentration (μg/g)	Soil moisture (%)	Soil sterility	Consortium inoclulation^*^	D (%) ± SD^**^	t_1/2_ (day)^***^	R^2****^	k^*****^ ±SE (day^−1^)	k^******^_(+/−)_± SE
FIP	15	100	Sterile	+	55.72^d^ ± 1.87	12	0.92	0.058 ± 0.005	1.28 ± 0.11
				−	51.34^d^ ± 3.10	14	0.97	0.048 ± 0.002	
			Non-sterile	+	71.34^bc^ ± 0.78	8	0.95	0.090 ± 0.005	1.22 ± 0.11
				−	65.00^c^ ± 6.68	9	0.95	0.074 ± 0.005	
		20	Sterile	+	53.90^d^ ± 2.74	13	0.98	0.055 ± 0.002	11.07 ± 0.94
				−	6.67^g^ ± 0.20	139	0.93	0.005 ± 0.000	
			Non-sterile	+	51.22^d^ ± 2.21	14	0.96	0.051 ± 0.003	2.00 ± 0.17
				−	30.21^e^ ± 1.38	27	0.94	0.026 ± 0.002	
	150	100	Sterile	+	69.93^bc^ ± 0.78	8	0.98	0.089 ± 0.003	6.88 ± 0.36
				−	16.66^f^ ± 0.54	54	0.98	0.013 ± 0.001	
			Non-sterile	+	89.27^a^ ± 1.10	4	0.98	0.156 ± 0.007	1.85 ± 0.09
				−	71.21^bc^ ± 1.95	8	0.99	0.084 ± 0.002	
		20	Sterile	+	68.44^bc^ ± 0.83	8	0.98	0.083 ± 0.003	13.09 ± 0.94
				−	8.62^fg^ ± 0.60	109	0.94	0.006 ± 0.000	
			Non-sterile	+	74.77^b^ ± 1.99	8	0.95	0.092 ± 0.006	3.05 ± 0.34
				−	33.92^e^ ± 0.93	23	0.90	0.030 ± 0.003	
THIO	15	100	Sterile	+	55.04^cde^ ± 1.85	12	0.97	0.060 ± 0.003	3.88 ± 0.20
				−	19.51^h^ ± 0.14	45	0.99	0.016 ± 0.000	
			Non-sterile	+	60.25^c^ ± 0.61	10	0.92	0.067 ± 0.005	1.25 ± 0.10
				−	53.36^de^ ± 0.65	13	0.99	0.054 ± 0.001	
		20	Sterile	+	36.10^f^ ± 2.20	23	0.97	0.031 ± 0.001	3.88 ± 0.43
				−	10.36^i^ ± 0.49	88	0.88	0.008 ± 0.001	
			Non-sterile	+	50.27^e^ ± 1.57	14	0.98	0.051 ± 0.002	2.13 ± 0.17
				−	27.63^g^ ± 1.90	29	0.93	0.024 ± 0.002	
	150	100	Sterile	+	74.40^b^ ± 3.08	7	0.98	0.106 ± 0.004	2.88 ± 0.12
				−	39.97^f^ ± 1.46	19	0.99	0.037 ± 0.001	
			Non-sterile	+	80.38^b^ ± 2.23	6	0.97	0.124 ± 0.006	0.75 ± 0.04
				−	89.34^a^ ± 0.32	4	0.99	0.166 ± 0.002	
		20	Sterile	+	56.45^cd^ ± 3.02	11	0.96	0.063 ± 0.003	6.38 ± 0.42
				−	12.56^i^ ± 0.28	70	0.98	0.010 ± 0.000	
			Non-sterile	+	79.01^b^ ± 0.62	5	0.87	0.139 ± 0.020	7.67 ± 1.18
				−	22.77^gh^ ± 1.09	38	0.96	0.018 ± 0.001	

SE: Standard error (*n* = 3). SD: Standard deviation (*n* = 3). ^*^ + and – indicate soils with and without consortium inoculation, respectively. ^**^Degradation after 14 days. Values with the same lowercase superscript letters in the column do not defer significantly (Tukey’s HSD post-hoc test, *p* = 0.05). ^***^Half-lives of FIP and THIO are calculated based on Eq. 2. ^****^The first-order exponential decay model (Eq. 1) coefficient of determination. ^*****^FIP and THIO degradation rates based on the first-order exponential decay model (Eq. 1). ^******^The contribution of the MD consortium to the degradation rates of FIP and THIO (Eq. 5). Standard errors are calculated via the error propagation method.

THIO degradation in soil by TD and MD consortia followed a parallel pattern, significantly increasing the degradation of THIO in both sterile and non-sterile soils at 20 and 100% moisture conditions (*p* < 0.01) (Tables 5, 6). In non-inoculated soils, the t_1/2_ of THIO in sterile and non-sterile samples ranged from 19 to 88 and 4 to 38 days, respectively. Inoculation with FD or MD consortia decreased the t_1/2_ of THIO to 7–23 and 5–14 days in sterile and non-sterile soils, respectively (Tables 5, 6). TD consortium, when inoculated, resulted in more pronounced THIO degradation at 20% moisture compared with 100% moisture in sterile soil at 15 μg/g (k_(+/−)_ = 4.5) and in both sterile and non-sterile soils at 150 μg/g (k_(+/−)_ = 5.42 and 4.61, respectively) (*p* < 0.01) (Table 6). The highest THIO degradation efficiency with the MD consortium was observed at 150 μg/g and 20% moisture in both sterile and non-sterile soils (k_(+/−)_ = 6.38 and 7.68, respectively; *p* < 0.01). At 15 μg/g, however, the increase in degradation was less pronounced (k_(+/−)_ = 3.88) with no significant difference between the two moisture contents (*p* > 0.05) (Table 5).

**Table 6:** Kinetics for THIO degradation in soil by the TD consortium.

Pesticide concentration (μg/g)	Soil moisture (%)	Soil sterility	Consortium inoclulation^*^	D (%) ± SD^**^	t_1/2_ (day)^***^	R^2****^	k^*****^ ± SE (day^−1^)	k^******^_(+/−)_ ± SE
15	100	Sterile	+	37.45^h^ ± 1.39	20	0.99	0.034 ± 0.001	2.32 ± 0.12
			−	18.57^i^ ± 1.38	47	0.97	0.015 ± 0.001	
		Non-sterile	+	56.90^de^ ± 1.10	11	0.98	0.063 ± 0.002	1.26 ± 0.05
			−	50.74^fg^ ± 0.65	14	0.99	0.050 ± 0.001	
	20	Sterile	+	47.17^g^ ± 1.30	16	0.99	0.044 ± 0.002	4.50 ± 0.38
			−	11.72^j^ ± 0.23	70	0.97	0.010 ± 0.001	
		Non-sterile	+	75.97^b^ ± 0.41	7	0.90	0.098 ± 0.002	1.51 ± 0.08
			−	61.68^d^ ± 2.53	11	0.99	0.065 ± 0.002	
150	100	Sterile	+	55.62^ef^ ± 1.85	12	0.99	0.056 ± 0.003	1.58 ± 0.10
			−	39.00^h^ ± 0.63	20	0.96	0.035 ± 0.001	
		Non-sterile	+	67.34^c^ ± 1.82	8	0.99	0.085 ± 0.005	0.98 ± 0.06
			−	69.80^c^ ± 1.70	8	0.95	0.086 ± 0.001	
	20	Sterile	+	54.75^ef^ ± 1.34	12	0.99	0.057 ± 0.003	5.42 ± 0.36
			−	13.90^ij^ ± 0.44	65	0.98	0.011 ± 0.001	
		Non-sterile	+	85.32^a^ ± 1.75	5	0.98	0.143 ± 0.004	4.61 ± 0.15
			−	34.77^h^ ± 0.07	22	0.99	0.031 ± 0.001	

SE: Standard error (*n* = 3). SD: Standard deviation (*n* = 3). ^*^ + and – indicate soils with and without consortium inoculation, respectively. ^**^Degradation after 14 days. Values with the same lowercase superscript letters in the column do not defer significantly (Tukey’s HSD post-hoc test, *p* = 0.05). ^***^Half-lives of THIO are calculated based on Eq. 2. ^****^The first-order exponential decay model (Eq. 1) coefficient of determination. ^*****^THIO degradation rates based on the first-order exponential decay model (Eq. 1). ^******^The contribution of the TD consortium to the degradation rates of THIO (Eq. 5). Standard errors are calculated via the error propagation method.

## 4 Discussion

### 4.1 Bacterial composition of the isolated consortia

In our investigation, three distinct consortia—FD, MD, and TD—were isolated to proficiently degrade FIP, THIO, and the FIP + THIO mixture, respectively. Each consortium exhibited a rich and diverse bacterial composition, unveiling the intricate microbial communities responsible for pesticide degradation. Notably, the MD consortium stood out with the highest Shannon diversity, surpassing FD and TD consortia. This elevated Shannon diversity in the MD consortium implies a greater overall diversity, encompassing both the variety of different taxa (richness) and their even distribution in abundance (Li et al., 2024). Conversely, MD and TD consortia displayed lower Faith’s_PD values than FD, indicating a relatively reduced phylogenetic diversity (Bai et al., 2023).

The prevalence of Proteobacteria, particularly *Azospirillum* sp. and Rhizobiales, across all consortia aligns with their well-established roles in soil ecosystems, encompassing functions such as nitrogen fixation, promotion of plant growth, and degradation of pesticides (Foster et al., 2004; Spain et al., 2009; Kaneko et al., 2010; Romeh and Hendawi, 2014; Liang et al., 2022; Degon et al., 2023). Furthermore, the presence of additional genera within Proteobacteria, such as *Ochrobactrum* sp., *Shinella* sp., *Sphingobium* sp., and *Sphingomonas* sp., underscores the potential of consortia for comprehensive pesticide degradation (Jiang et al., 2011; Wu et al., 2016; Vanitha et al., 2023; Wang et al., 2023).

*Chitinophaga* sp. and *Bacteroides* sp., identified within the Bacteroidetes phylum, are recognized degraders of carbofuran and atrazine, highlighting their significant ecological roles within the consortia (Karpouzas et al., 2000; Huang et al., 2020). The presence of anaerobic bacteria, including *Oscillospira* sp. and *Dorea* sp. and genera of Ruminococcaceae, Lachnospiraceae, and Clostridiales, suggests a potential contribution to reductive/anaerobic reactions in the soil, thereby facilitating xenobiotic degradation (Huang et al., 2019; Lu et al., 2019). Finally, the dominance of Actinobacteria, specifically *Bifidobacterium* sp., *Mycobacterium* sp., and *Acinetobacter* sp., within our consortia is noteworthy. These populations, typically found in the rhizosphere, are well-documented pesticide degraders, contributing to the consortia’s efficacy in pesticide degradation (Teixeira et al., 2010; Kandil et al., 2015; Zhan et al., 2018; Kumar et al., 2021). The intricate interplay of these diverse bacterial populations within each consortium underscores their potential for comprehensive pesticide remediation in various environmental contexts.

### 4.2 FIP and THIO degradation with the consortia

Our study demonstrated the capability of all three consortia to effectively degrade pesticides across a concentration range of 25–800 μg/mL in culture medium. However, a noteworthy trend emerged, indicating a reduction in degradation rates at higher pesticide concentrations. While microbial tolerance to pesticides has been documented in previous studies (Yang et al., 2018; Zhang et al., 2020) due to repeated exposure during enrichment cycles, research has also revealed a detrimental correlation between high pesticide concentrations and the degradation capacity of microbial isolates or consortia. This correlation can be attributed to impaired growth, metabolism, and enzyme activity of microorganisms at higher pesticide concentrations (Li et al., 2020; Zhang et al., 2020; Bhatt et al., 2021a; Li H. et al., 2022; Pang et al., 2023).

In our investigation, the RSM approach, coupled with the Box–Behnken design, a widely acknowledged technique for optimizing degradation conditions of pesticides by microbial consortia, was employed. By considering key growth-determining factors such as culture pH, pesticide concentration, and inoculum size, our research aimed to identify conditions that would maximize the consortia’s pesticide degradation capabilities (Zhan et al., 2018; Birolli et al., 2020; Bhatt et al., 2021a,b; Pang et al., 2023). Notably, our findings unveiled that an inoculum size of 4–6%, along with conditions favoring a neutral to alkaline pH range, enhanced the degradation ability activity of the consortia. Furthermore, pesticide concentrations within the range of 60–100 μg/mL were identified as providing an optimal energy source for the consortia without inducing toxic effects (Zhan et al., 2018; Li H. et al., 2022; Bhatt et al., 2023; Pang et al., 2023).

### 4.3 Metabolic pathway of FIP and THIO degradation with the consortia

In this study, metabolic pathways involved in FIP and THIO degradation by the isolated consortia were unraveled. FD and MD consortia exhibited a shared initial step in FIP degradation through the hydrolytic cleavage of the C-N bond, which was succeeded by subsequent oxidation and hydrolysis reactions. These results align with the existing literature, highlighting oxidative and hydrolytic processes as primary degradation pathways for FIP when microbial isolates are employed (Mandal et al., 2013, 2014). Notable compounds resembling our findings, such as octadecane sulfonyl, hexadecane-1-sulfonic acid, 4-hydroxy-, delta-sultone, and 5,8,11-heptadecatrienyl methyl ester, have been identified during FIP degradation by *Bacillus* sp. and *Streptomyces rochei* (Abraham and Gajendiran, 2019; Bhatt et al., 2021a). Another metabolite observed, 1,2-benzene dicarboxylic acid, with the potential to transform into 1,4-benzenediol, further reinforces the diversity of degradation pathways in FIP breakdown (Abraham and Gajendiran, 2019; Bhatt et al., 2021b). It is intriguing to note that our study did not detect FIP sulfone, a known early metabolite (Uniyal et al., 2016; Bhatt et al., 2021a,b), likely due to its rapid production during degradation, while our measurements were conducted after a 10-day incubation period.

Turning to the degradation pathways of THIO by the TD and MD consortia, the process involved the cleavage of the C-S bond, resulting in the production of benzenecarbothioic acid, S-methyl ester, and subsequent compounds such as carbamothioic acid, diethyl-, S-ethyl ester, benzothiazole, 2-methyl-, and 1-hexadecanethiol. These results correspond well with the findings by Chu et al. (2017), who identified diethylcarbamothioic S-acid as the primary product in aerobic THIO degradation by *Acidovorax* sp. Moreover, metabolites such as S-4-chlorobenzyl ethylthiocarbamate and 4-chlorobenzyl mercaptan were observed in the degradation of THIO by *Pseudomonas* sp. and *Cupriavidus oxalaticus* (Duc, 2023; Duc et al., 2023), which further corroborated our proposed pathways.

### 4.4 Soil bioaugmentation test with the isolated consortia

To comprehensively evaluate the degradative potential of FD, TD, and MD consortia in paddy soils, extensive bioaugmentation tests were conducted. Our key findings can be summarized as follows: (a) Across diverse conditions of pesticide concentration, soil moisture, and sterility, all consortia significantly enhanced the degradation of FIP and THIO compared with non-inoculated soils, (b) notably, the consortia exhibited heightened degradation efficiencies in sterile soils, where the absence of indigenous biodegradation processes allowed them to serve as primary agents responsible for pesticide degradation, and (c) optimal degradation of FIP and THIO was observed at 20% moisture conditions, surpassing efficiencies observed at 100% moisture.

The increased efficacy of the consortia in sterile soils underscores their ability to drive biodegradation processes in environments, otherwise limited in such activities. Even in non-sterile soils with existing microbial communities, the consortia demonstrated significant impacts on pesticide degradation. Although degradation rates were slightly lower compared with sterile soils, the consortia’s effectiveness suggests potential synergistic interactions with the native microbial community, warranting further exploration of niche differentiation or cooperative metabolic activities. Our findings align with the study by Pang et al. (2023) and Bhatt et al. (2021b), indicating that introducing bacterial consortia enhances pesticide degradation. However, our study uniquely delves into the role of sterile soil conditions, shedding light on the consortia’s capacity to establish and function in the absence of competing microorganisms.

Furthermore, the superior degradation efficiency of our consortia in low moisture content can be attributed to the aerobic nature of dominant bacterial populations. Even under dominant anoxic conditions in soils with 100% moisture, our consortia remained significantly effective, emphasizing the role of anaerobic bacterial populations such as *Oscillospira* sp. and *Dorea* sp. and genera of Ruminococcaceae, Lachnospiraceae, and Clostridiales in the degradation of FIP and THIO. Previous reports on increased abundance and activity of obligate anaerobic bacteria such as Clostridiales under low oxygen conditions in flooded soils support these results (Zhou et al., 2012; Lauga et al., 2013; Elsayed et al., 2015; Torabi et al., 2020). This promising outcome suggests that the high species richness of bacteria in FD, TD, and MD consortia endows them with efficient adaptability and biodegradability across varied soil conditions (Bhatt et al., 2021c; Lin et al., 2022).

## 5 Conclusion

In this study, three bacterial consortia, namely, FD, TD, and MD, were successfully isolated from paddy soils using the enrichment culture method and characterized. These consortia demonstrated a remarkable capacity for degrading specific pesticides: FIP, THIO, and the FP + THIO mixture, respectively. The efficiency of pesticide degradation by all consortia exceeded 80%, accomplished within 10 days.

The bacterial populations within the consortia exhibited considerable diversity, with predominant genera identified as *Azospirillum*, *Ochrobactrum*, *Sphingobium*, and *Sphingomonas*. Notably, these consortia displayed robust tolerance and degradation capabilities even when exposed to high pesticide concentrations, reaching up to 800 μg/mL for both FIP and THIO. The degradation mechanisms employed by the consortia primarily involved oxidative and hydrolytic reactions, representing a novel pathway for the degradation of FIP and THIO.

Comprehensive bioaugmentation tests were conducted in soils contaminated with FIP and THIO to further evaluate their practical applicability. Consortia consistently demonstrated substantial pesticide degradation abilities in diverse conditions, including various concentrations, soil moistures, and sterile or non-sterile soil conditions.

In conclusion, the findings from this research highlight the promising potential of FD, TD, and MD consortia for bioremediation and bioaugmentation of FIP and THIO in polluted paddy soils. The remarkable pesticide-degrading capabilities, coupled with their adaptability to varying soil conditions, position these bacterial consortia as valuable tools for addressing pesticide contamination in agricultural ecosystems. The application of FD, TD, and MD consortia in bioremediation strategies holds promise for enhancing the sustainability and environmental health of paddy soil ecosystems.

## Data availability statement

The raw sequences used in this study are deposited in the BioProject database (http://www.ncbi.nlm.nih.gov/bioproject/1061744), accession number PRJNA1061744. Additional generated datasets are included in the article/supplementary material.

## Author contributions

NF: Data curation, Investigation, Methodology, Writing – original draft. ET: Conceptualization, Data curation, Formal analysis, Funding acquisition, Methodology, Project administration, Software, Supervision, Writing – review & editing. AP: Investigation, Methodology, Writing – review & editing. EO: Investigation, Methodology, Writing – review & editing. KT: Investigation, Methodology, Writing – review & editing.
