# Supplementary figures and images for: Efficacy of novel bacterial consortia in degrading fipronil and thiobencarb in paddy soil: a survey for community structure and metabolic pathways
Source: Front Microbiol. 2024 May 15;15:1366951. doi: 10.3389/fmicb.2024.1366951 (PMC11133635; doi:10.3389/fmicb.2024.1366951)

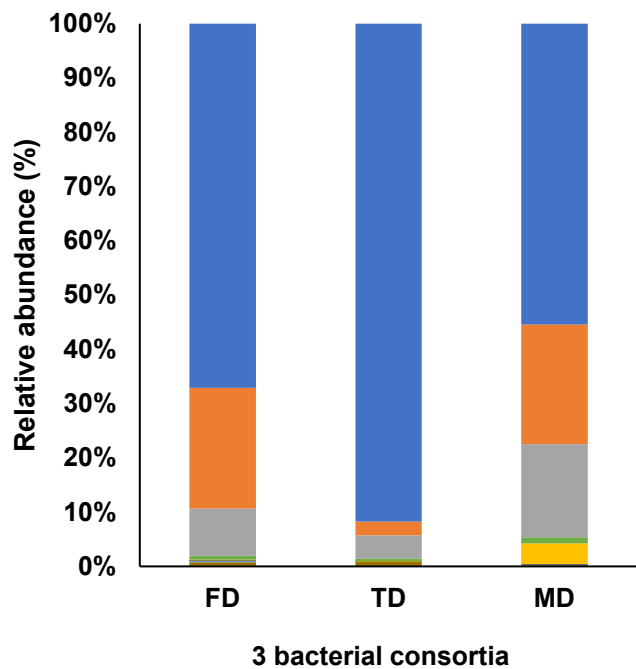

**Fipronil and Thiobenacarb degradation**

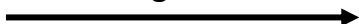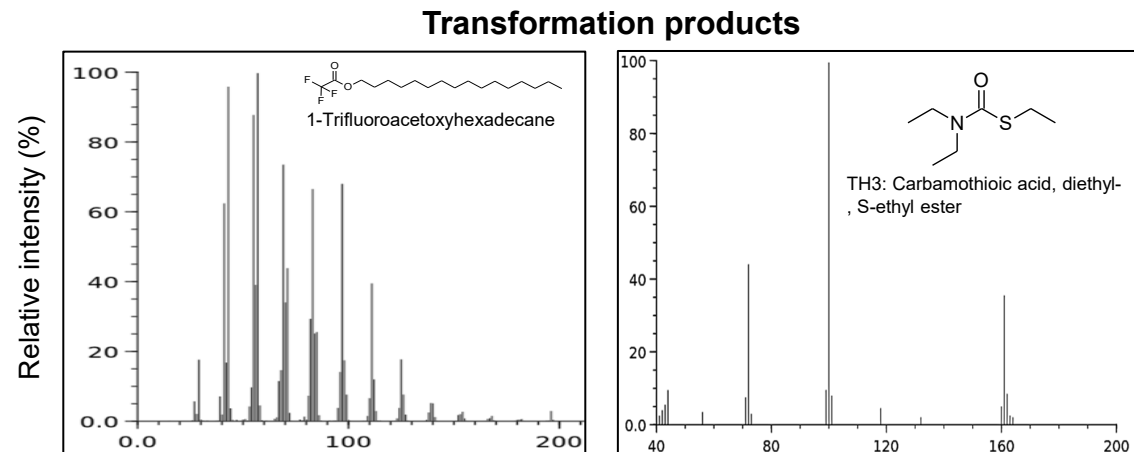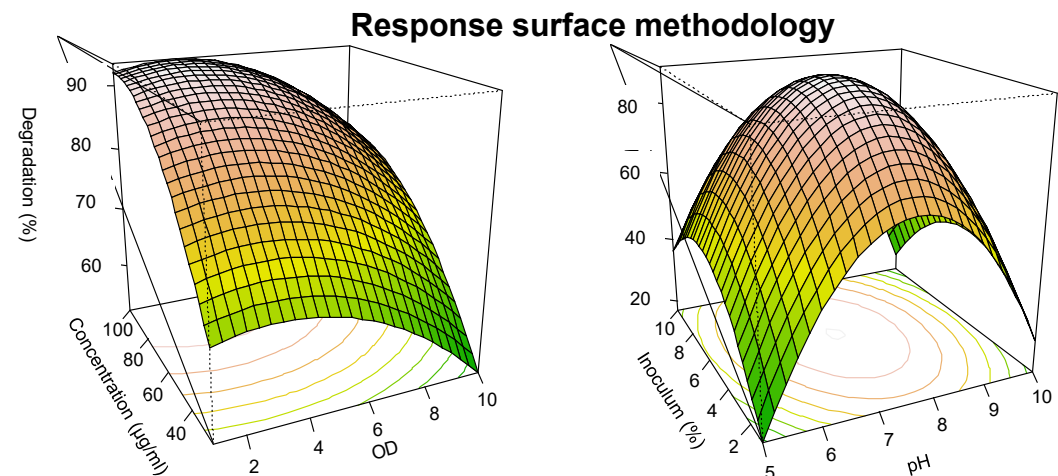

**Soil bioaugmentation tests**

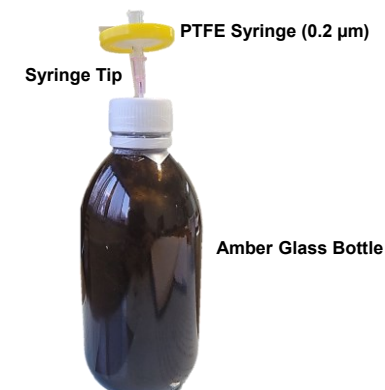

Supplement: Supplementary file 1 [file Data_Sheet_1.pdf]
