# Supplementary material for: Efficacy of novel bacterial consortia in degrading fipronil and thiobencarb in paddy soil: a survey for community structure and metabolic pathways
Source: Front Microbiol. 2024 May 15;15:1366951. doi: 10.3389/fmicb.2024.1366951 (PMC11133635; doi:10.3389/fmicb.2024.1366951)
Supplement: Supplementary file 2 [file Data_Sheet_2.docx]

***Supplementary Material***

**Efficacy of novel bacterial consortia in degrading fipronil and thiobencarb in paddy soil: a survey for community structure and metabolic pathways**

**Nastaran Faridy^1^, Ehssan Torabi^1*^, Ahmad Ali Pourbabaee^2^, Ebrahim Osdaghi^1^, Khalil Talebi^1^**

^1^Department of Plant Protection, Faculty of Agriculture, College of Agriculture and Natural Resources, University of Tehran, Karaj. Iran.

^2^Department of Soil Science, Faculty of Agriculture, College of Agriculture and Natural Resources, University of Tehran, Karaj. Iran.

*** Correspondence:**Ehssan Torabi

eh_torabi@ut.ac.ir

**Section A. Physicochemical properties of pesticides used in the study**

Supplementary Table 1 Physicochemical properties of FIP, THIO, and CART

| Properties^*^ | FIP | THIO |
| --- | --- | --- |
| IUPAC name | 5-amino-1-[2,6-dichloro-4-(trifluoromethyl)phenyl]-4-(trifluoromethylsulfinyl)pyrazole-3-carbonitrile | S-4-chlorobenzyl diethyl(thiocarbamate) |
| pK_a_ | Not applicable | Not applicable |
| Molecular mass | 437.1 | 257.78 |
| Solubility in water (mg/L^-^) | 3.78 | 28 |
| Vapor Pressure (mPa) | 0.002 | 2.39 |
| Log K_ow_ | 3.75 | 4.23 |
| Henry's Law constant (Pa m³/mol) | 2.31×10^-4^ | 3.68 × 10^-2^ |
| K_oc_ | 825 to 6863 | 309-5000 |
| Hydrolysis | Stable to hydrolysis at pH 5.5 and pH 7, but has a hydrolysis half-life of 28 days at pH 9 | Hydrolysis half-life >1 year (25 °C, pH 4,7, and 9). |

**^*^** Values from PPDB <http://sitem.herts.ac.uk/aeru/ppdb/>

**Section B. Collection of soil samples**

Soil samples for the consortia isolation process and degradation tests were collected during 2020 and 2021 from a paddy field located in the city of Amol, Mazandaran province, Iran (Longitude: 52°27'54.58''E, Latitude: 36°228'45.14''N, Height: 30 m).

From the field, 4 samples were collected diagonally from the upper soil layer (0 – 30 cm), and a final amount of 10 kg was selected, transferred to the laboratory, and kept at 4 °C. Samples were mixed, homogenized, and sieved (2 mm mesh) for analysis of their physicochemical properties. Physical and chemical properties including texture, bulk density (BD), organic carbon (OC), pH (in water), and cation exchange capacity (CEC) were analyzed according to ISO standard procedures (http://www.iso.org). Soil macro/microelements amounts were measured using atomic absorption spectrophotometry (ICP-AES, ICAP6500, Thermo Fisher Scientific) (Babcsányi et al., 2014). Soil pH was assayed from a soil/distilled water ratio of 1:2.5 (*w/v*) with a pH meter (Singh et al., 2003). The soil's electrical conductivity (EC) was analyzed based on the Singh et al. (2003) procedure using the saturated soil extract. Soil field capacity (FC) was measured based on the method of Karpouzas et al. (2004) by saturating and draining the soil for 24 h. Total nitrogen (N_tot_) was detected using a Vario MAX CNS elemental analyzer. A summary of soil physicochemical properties is listed in Supplementary Table 2.

Supplementary Table 2. Soil physicochemical properties

| Soil texture (USDA classification) | Silty Clay loam |
| --- | --- |
| Sand (%) | 16 (4)^*^ |
| Clay (%) | 34(6) |
| Silt (%) | 50 (3) |
| BD (g/cm3) | 0.94 (0.05) |
| pH (in water) | 8.3 (0.6) |
| EC (ds/m) | 1.27 (0.05) |
| OC (%) | 2.91 (0.4) |
| N_tot_ (%) | 0.32 (0.06) |
| P (mg/kg) | 5.53 (0.4) |
| K (mg/kg) | 88 (7) |
| Fe (mg/kg) | 63.7 (8.9) |
| Zn (mg/kg) | 2.34 (0.07) |
| Mn (mg/kg) | 8.22 (0.8) |
| Cu (mg/kg) | 2.8 (0.05) |
| Na (me/L) | 1.88 (0.2) |
| Ca (me/L) | 8.2 (0.3) |
| Mg (me/L) | 3.4 (0.04) |
| CEC (%) | 18.1 (2.4) |
| FC (%) | 78 (6) |

**^a:^** Means of triplicated with standard deviations in parentheses; **BD**: bulk density; **OC**: organic carbon; **N_tot_**: toral nitrogen; **EC**: electrical conductivity; **CEC**: cation exchange capacity; **FC**: field capacity

**Section C. Degradation efficiencies of FD, TD, and MD consortia**

Supplementary Table 3. Degradation efficiencies of FD, TD, and MD consortia

| Time (day) | Pesticide concentration (µg/ml) | Degradation (%) ± SD | | | |
| --- | --- | --- | --- | --- | --- |
|  |  | FIP  (FD consortium) | THIO  (TD consortium) | FIP  (MD consortium) | THIO  (MD consortium) |
| 0 | 25 | 0.0 ± 2.2 | 0.0 ± 9.4 | 0.0 ± 3.2 | 0.0 ± 0.2 |
|  | 50 | 0.0 ± 0.4 | 0.0 ± 10.2 | 0.0 ± 1.3 | 0.0 ± 0.7 |
|  | 100 | 0.0 ± 0.1 | 0.0 ± 2.2 | 0.0 ± 3.2 | 0.0 ± 1.5 |
|  | 200 | 0.0 ± 0.9 | 0.0 ± 0.3 | 0.0 ± 16.8 | 0.0 ± 5.8 |
|  | 400 | 0.0 ± 0.5 | 0.0 ± 2.8 | 0.0 ± 1.3 | 0.0 ± 0.7 |
|  | 800 | 0.0 ± 9.0 | 0.0 ± 1.8 | 0.0 ± 4.2 | 0.0 ± 1.0 |
|  | Control | 0.0 ± 4.9 | 0.0 ± 3.9 | 0.0 ± 2.0 | 0.0 ± 0.1 |
| 1 | 25 | 24.2 ± 0.3 | 18.1 ± 1.2 | 36.9 ± 2.0 | 36.3 ± 0.5 |
|  | 50 | 23.0 ± 1.0 | 22.6 ± 0.6 | 47.2 ± 0.2 | 48.3 ± 5.8 |
|  | 100 | 19.6 ± 1.0 | 16.7 ± 1.0 | 47.0 ± 0.9 | 32.1 ± 2.3 |
|  | 200 | 20.6 ± 1.1 | 15.7 ± 1.4 | 38.8 ± 1.1 | 13.1 ± 14.1 |
|  | 400 | 14.2 ± 2.0 | 14.3 ± 1.4 | 24.1 ± 11.1 | 20.7 ± 0.7 |
|  | 800 | 12.8 ± 0.4 | 27.8 ± 0.7 | 39.2 ± 0.5 | 7.0 ± 1.0 |
|  | Control | 3.3 ± 0.5 | 3.0 ± 0.5 | 8.6 ± 0.9 | 1.7 ± 0.4 |
| 3 | 25 | 57.0 ± 0.5 | 45.3 ± 0.3 | 48.6 ± 1.2 | 74.6 ± 0.9 |
|  | 50 | 54.4 ± 1.0 | 49.2 ± 0.6 | 58.4 ± 1.4 | 61.1 ± 11.4 |
|  | 100 | 48.2 ± 0.6 | 41.6 ± 0.6 | 53.9 ± 1.8 | 54.6 ± 1.1 |
|  | 200 | 47.5 ± 1.5 | 40.1 ± 0.7 | 49.7 ± 0.9 | 41.4 ± 1.4 |
|  | 400 | 36.1 ± 1.1 | 39.2 ± 0.8 | 34.3 ± 1.1 | 41.4 ± 1.2 |
|  | 800 | 33.2 ± 0.6 | 37.0 ± 0.8 | 42.5 ± 0.9 | 23.3 ± 1.6 |
|  | Control | 7.6 ± 0.6 | 8.1 ± 0.3 | 17.3 ± 1.3 | 3.5 ± 1.5 |
| 5 | 25 | 73.2 ± 3.5 | 63.4 ± 0.5 | 64.3 ± 2.1 | 89.9 ± 0.2 |
|  | 50 | 72.6 ± 0.2 | 66.4 ± 1.0 | 72.2 ± 1.5 | 77.7 ± 0.4 |
|  | 100 | 66.4 ± 0.2 | 61.1 ± 1.7 | 69.3 ± 0.7 | 68.9 ± 1.0 |
|  | 200 | 62.6 ± 0.4 | 57.7 ± 0.3 | 61.4 ± 0.8 | 55.9 ± 1.9 |
|  | 400 | 52.5 ± 0.2 | 48.4 ± 1.4 | 48.8 ± 1.2 | 52.6 ± 0.7 |
|  | 800 | 49.0 ± 0.2 | 42.0 ± 2.6 | 49.2 ± 0.9 | 42.6 ± 0.3 |
|  | Control | 11.5 ± 1.5 | 14.9 ± 1.3 | 24.5 ± 2.1 | 9.4 ± 0.8 |
| 10 | 25 | 94.1 ± 0.3 | 86.6 ± 0.4 | 80.6 ± 1.4 | 97.1 ± 0.1 |
|  | 50 | 92.0 ± 0.4 | 87.9 ± 0.5 | 87.9 ± 1.1 | 89.0 ± 1.0 |
|  | 100 | 89.6 ± 0.8 | 80.1 ± 2.0 | 89.1 ± 0.5 | 86.8 ± 0.2 |
|  | 200 | 87.0 ± 0.4 | 82.0 ± 0.7 | 78.1 ± 0.4 | 77.7 ± 1.1 |
|  | 400 | 77.5 ± 0.9 | 91.5 ± 0.7 | 74.9 ± 0.9 | 69.4 ± 1.7 |
|  | 800 | 74.1 ± 0.6 | 60.3 ± 1.1 | 66.7 ± 0.3 | 63.2 ± 3.0 |
|  | Control | 20.1 ± 0.6 | 24.6 ± 0.5 | 39.0 ± 1.5 | 18.8 ± 0.4 |

**Section D. Box-Behnken experimental design**

Supplementary Table 4. Box-Behnken experimental design and the response of the dependent variable for FIP and THIO degradation with FD, TD, and MD consortia

| Experiment No. | Factors | | | Degradation (%) | | | |
| --- | --- | --- | --- | --- | --- | --- | --- |
|  | X_1_ | X_2_ | X_3_ | FIP  (FD consortium) | THIO  (TD consortium) | FIP  (MD consortium) | THIO  (MD consortium) |
| 1 | 7 | 50 | 5 | 78.6 | 83.9 | 87.1 | 84.9 |
| 2 | 5 | 100 | 5 | 59.5 | 78.0 | 24.7 | 11.1 |
| 3 | 7 | 50 | 5 | 83.7 | 81.0 | 88.9 | 85.9 |
| 4 | 7 | 25 | 1 | 65.8 | 44.6 | 40.5 | 47.8 |
| 5 | 7 | 50 | 5 | 84.1 | 82.5 | 88.7 | 82.5 |
| 6 | 10 | 25 | 5 | 39.5 | 18.7 | 18.4 | 15.9 |
| 7 | 7 | 100 | 10 | 79.5 | 47.5 | 58.9 | 58.3 |
| 8 | 7 | 25 | 10 | 46.6 | 42.6 | 56.2 | 41.8 |
| 9 | 5 | 50 | 10 | 39.8 | 13.6 | 33.0 | 19.2 |
| 10 | 10 | 100 | 5 | 64.4 | 26.4 | 48.7 | 39.6 |
| 11 | 7 | 100 | 1 | 96.4 | 75.0 | 79.0 | 45.9 |
| 12 | 5 | 50 | 1 | 57.7 | 40.7 | 5.1 | 3.8 |
| 13 | 7 | 50 | 5 | 78.3 | 75.4 | 85.0 | 83.6 |
| 14 | 5 | 25 | 5 | 40.6 | 1.7 | 59.0 | 2.0 |
| 15 | 7 | 50 | 5 | 82.2 | 87.7 | 89.1 | 84.9 |
| 16 | 10 | 50 | 10 | 45.9 | 2.6 | 31.3 | 14.6 |
| 17 | 10 | 50 | 1 | 41.8 | 13.3 | 32.1 | 19.2 |

**X_1_:** Culture pH**, X_2_:** pesticide concentration (µg/ml)**, X_3_:** Inoculum size (%). Each experiment was conducted in triplicates.

**Section E. Soil degradation tests of FIP and THIO by the isolated consortia**

Supplementary Table 5. Summary of soil degradation experimental design

| Microcosms | Consortia | Pesticide | Pesticide concentration in soil (µg/g) | Soil sterility* | Consortia inoculation | Number of replicates | Incubation period (day) |
| --- | --- | --- | --- | --- | --- | --- | --- |
| 1 | FD | FIP | 15 | Sterile | Inoculated | 3 | 14 |
| 2 |  |  |  |  | Uninoculated | 3 | 14 |
| 3 |  |  |  | Non-sterile | Inoculated | 3 | 14 |
| 4 |  |  |  |  | Uninoculated | 3 | 14 |
| 5 |  |  | 150 | Sterile | Inoculated | 3 | 14 |
| 6 |  |  |  |  | Uninoculated | 3 | 14 |
| 7 |  |  |  | Non-sterile | Inoculated | 3 | 14 |
| 8 |  |  |  |  | Uninoculated | 3 | 14 |
| 9 | TD | THIO | 15 | Sterile | Inoculated | 3 | 14 |
| 10 |  |  |  |  | Uninoculated | 3 | 14 |
| 11 |  |  |  | Non-sterile | Inoculated | 3 | 14 |
| 12 |  |  |  |  | Uninoculated | 3 | 14 |
| 13 |  |  | 150 | Sterile | Inoculated | 3 | 14 |
| 14 |  |  |  |  | Uninoculated | 3 | 14 |
| 15 |  |  |  | Non-sterile | Inoculated | 3 | 14 |
| 16 |  |  |  |  | Uninoculated | 3 | 14 |
| 17 | MD | FIP + THIO | 15 | Sterile | Inoculated | 3 | 14 |
| 18 |  |  |  |  | Uninoculated | 3 | 14 |
| 19 |  |  |  | Non-sterile | Inoculated | 3 | 14 |
| 20 |  |  |  |  | Uninoculated | 3 | 14 |
| 21 |  |  | 150 | Sterile | Inoculated | 3 | 14 |
| 22 |  |  |  |  | Uninoculated | 3 | 14 |
| 23 |  |  |  | Non-sterile | Inoculated | 3 | 14 |
| 24 |  |  |  |  | Uninoculated | 3 | 14 |

^*^ Soils were sterilized by three times autoclaving at 121 °C for 20 min (Berns et al., 2008).

**Section F. Method validation for FIP and THIO analysis**

**Precision and accuracy assessment**

The precision and accuracy of each extraction method were evaluated by computing the recoveries and relative standard deviations (RSDs) of FIP and THIO spiked into pesticide-free soils (at two moisture contents of 20% and 100%) or mineral salt media (MSM), as described below (SANTE, 2021). For this, 20 g of soil or 10 ml of MSM were spiked with aqueous solutions of FIP and THIO in triplicate. The spiking concentrations were 0.2, 1, and 2 µg/g or µg/mL

**Detection limits and linearity assessment**

The slope (m) and root mean square error (RMSE) of the solvent calibration curve (in MeOH) were employed to ascertain the instrumental detection limit (IDL) and IQL, respectively, employing Eqs. 1 and 2 (Corley, 2003).

$IDL \left( \mu g/mL \right)=\frac{3\times RMSE}{m}$ (1)

$IQL \left( \mu g/mL \right)=\frac{10\times RMSE}{m}$ (2)

The estimated method detection (EMDL) and quantification limits were measured according to Eqs. 3 and 4, respectively (Singh et al., 2007):

$EMDL \left( \mu g/mL \right)=\frac{IDL\times V\times100}{\%Recovery\times M}$ (3)

$EMQL \left( \mu g/mL \right)=\frac{IQL\times V\times100}{\%Recovery\times M}$ (4)

V and M indicate the solution volume used for analysis (measured in mL) and the amount of soil used for extraction (measured in g).

Linearity was assessed through matrix-matched calibration curves established in blank soil/MSM extracts, encompassing five concentrations of each pesticide ranging from EMQL to 10 EMQL. Results of the method validation procedure are presented in Supplementary Table 5.

Supplementary Table 6 Method validation results for FIP and THIO analysis in soil and MSM cultures

| Pesticide | Matrix Type | Spiked concentrations  (µg/g) (µg/mL) | Recovery (%) | RSD (%) | Linearity  (Matrix-matched calibration curve) | | | EMDL  (µg/g) (µg/mL) | EMQL  (µg/g) (µg/mL) | IDL  (µg/mL) | IQL  (µg/mL) |
| --- | --- | --- | --- | --- | --- | --- | --- | --- | --- | --- | --- |
|  |  |  |  |  | Range  (µg/g) (µg/mL) | Curve equation | R^2^ |  |  |  |  |
| FIP | Soil (20% v*/w* moisture) | 0.2 | 90.42 | 1.39 | 0.18 - 1.80 | Y = 18340X + 2107 | 0.96 | 0.05 | 0.18 | 1.14 | 3.81 |
|  |  | 1 | 94.33 | 2.18 |  |  |  |  |  |  |  |
|  |  | 2 | 93.68 | 1.69 |  |  |  |  |  |  |  |
|  | Soil (100% *v/w* moisture) | 0.2 | 109.42 | 1.83 | 0.18 - 1.80 | Y = 24171X - 540 | 0.97 | 0.06 | 0.18 |  |  |
|  |  | 1 | 100.33 | 2.25 |  |  |  |  |  |  |  |
|  |  | 2 | 99.55 | 1.61 |  |  |  |  |  |  |  |
|  | MSM culture | 2 | 99.45 | 7.63 | 0.08 – 0.8 | Y = 25712X + 14 | 0.99 | 0.02 | 0.08 |  |  |
|  |  | 5 | 91.25 | 9.54 |  |  |  |  |  |  |  |
|  |  | 10 | 98.36 | 4.23 |  |  |  |  |  |  |  |
| THIO | Soil (20% v*/w* moisture) | 0.2 | 91.42 | 2.41 | 0.14 – 1.40 | Y = 89526X+ 10650 | 0.96 | 0.04 | 0.14 | 0.73 | 2.44 |
|  |  | 1 | 85.54 | 1.75 |  |  |  |  |  |  |  |
|  |  | 2 | 89.47 | 3.21 |  |  |  |  |  |  |  |
|  | Soil (100% *v/w* moisture) | 0.2 | 115.42 | 2.75 | 0.11 – 1.1 | Y = 54911X+ 9402 | 0.90 | 0.03 | 0.11 |  |  |
|  |  | 1 | 106.25 | 3.41 |  |  |  |  |  |  |  |
|  |  | 2 | 112.08 | 1.45 |  |  |  |  |  |  |  |
|  | MSM culture | 0.2 | 98.78 | 4.31 | 0.05 - 0.5 | Y = 89462X+ 7157 | 0.93 | 0.01 | 0.05 |  |  |
|  |  | 1 | 97.65 | 5.24 |  |  |  |  |  |  |  |
|  |  | 2 | 92.25 | 8.78 |  |  |  |  |  |  |  |

**FIP and THIO**: fipronil and thiobencarb respectively **RSD**: relative standard deviation of triplicates; **IDL**: instrumental detection limit; **IQL**: instrumental quantification limit; **EMDL**: estimated method detection limit; **EMQL**: estimated method quantification limit

**Section G. Relative abundances of main bacterial phyla present in the isolated consortia at the genus level**

Supplementary Figure 1. Relative abundance of Proteobacteria in the isolated consortia at genus level.

Supplementary Figure 2. Relative abundance of Bacteroidetes in the isolated consortia at genus level.

Supplementary Figure 3. Relative abundance of Firmicutes in the isolated consortia at genus level.

Supplementary Figure 4. Relative abundance of Actinobacteria in the isolated consortia at genus level.

**Section H: Mass spectra of FIP and THIO metabolites**

Supplementary Figure 5. Mass spectra and chemical structures of metabolites identified through FIP degradation by FD and MD consortia.

Supplementary Figure 6. Mass spectra and chemical structures of metabolites identified through THIO degradation by TD and MD consortia.

**References**

Babcsányi, I., Imfeld, G., Granet, M., and Chabaux F. (2014). Copper stable isotopes to trace copper behavior in wetland systems. *Environ. Sci. Technol.* 48, 5520–5529. doi: 10.1021/es405688v

Berns, A.E., Philipp, H., Narres, H.D., Burauel, P., Vereecken, H., and Tappe, W. (2008). Effect of gamma‐sterilization and autoclaving on soil organic matter structure as studied by solid state NMR, UV and fluorescence spectroscopy. *Eur. J. Soil. Sci.* 59, 540-550. doi: 10.1111/j.1365-2389.2008.01016.

Corley J. (2003). “Best practices in establishing detection and quantification limits for pesticide residues in foods” in Handbook of Residue Analytical Methods for agrochemicals, ed. P.W. Lee, H. Aizawa, A. C. Barefoot, J. J. Murphy (Wiley, Chichester), 59–75

Karpouzas, D. G., Hatziapostolou, P., Papadopoulou‐Mourkidou, E., Giannakou, I.O., & Georgiadou, A. (2004). The enhanced biodegradation of fenamiphos in soils from previously treated sites and the effect of soil fumigants. *Environ. Toxicol. Chem.* 23, 2099-2107. doi: 10.1897/03-531

SANTE (2021). Analytical quality control and method validation procedures for pesticide residues analysis in food and feed SANTE 11312/2021. Retrieved August 5, 2023, from <https://www.accredia.it/en/documento/guidance-sante-11312-2021-analytical-quality-control-and-method-validation-procedures-for-pesticide-residues-analysis-in-food-and-feed/>

Singh, B.K., Walker, A., Morgan, J.A.W., & Wright, D.J. (2003). Role of soil pH in the development of enhanced biodegradation of fenamiphos. *Appl. Environ. Microbiol*. 69, 7035-7043. doi: 10.1128%2FAEM.69.12.7035-7043.2003

Singh, S. B., Foster, G. D., and Khan, S. U. (2007). Determination of thiophanate methyl and carbendazim residues in vegetable samples using microwave-assisted extraction. *J. Chromatogr. A* 1148, 152-157. doi: 0.1016/j.chroma.2007.03.019
